# Genome-wide association studies in apple reveal loci of large effect controlling apple polyphenols

**DOI:** 10.1038/s41438-019-0190-y

**Published:** 2019-09-07

**Authors:** Kendra A. McClure, YuiHui Gong, Jun Song, Melinda Vinqvist-Tymchuk, Leslie Campbell Palmer, Lihua Fan, Karen Burgher-MacLellan, ZhaoQi Zhang, Jean-Marc Celton, Charles F. Forney, Zoë Migicovsky, Sean Myles

**Affiliations:** 1 Department of Plant and Animal Sciences, Faculty of Agriculture, Dalhousie University, Truro, NS B2N 5E3 Canada; 2 Agriculture and Agri-Food Canada, Kentville Research and Development Centre, Kentville, NS B4N 1J5 Canada; 3 College of Horticulture, South China Agriculture University, Guangzhou, 510642 China; 4 IRHS, Agrocampus-Ouest, INRA, Université d’Angers, SFR 4207 QuaSaV, Beaucouzé, France

**Keywords:** Plant breeding, Secondary metabolism

## Abstract

Apples are a nutritious food source with significant amounts of polyphenols that contribute to human health and wellbeing, primarily as dietary antioxidants. Although numerous pre- and post-harvest factors can affect the composition of polyphenols in apples, genetics is presumed to play a major role because polyphenol concentration varies dramatically among apple cultivars. Here we investigated the genetic architecture of apple polyphenols by combining high performance liquid chromatography (HPLC) data with ~100,000 single nucleotide polymorphisms (SNPs) from two diverse apple populations. We found that polyphenols can vary in concentration by up to two orders of magnitude across cultivars, and that this dramatic variation was often predictable using genetic markers and frequently controlled by a small number of large effect genetic loci. Using GWAS, we identified candidate genes for the production of quercitrin, epicatechin, catechin, chlorogenic acid, 4-*O*-caffeoylquinic acid and procyanidins B1, B2, and C1. Our observation that a relatively simple genetic architecture underlies the dramatic variation of key polyphenols in apples suggests that breeders may be able to improve the nutritional value of apples through marker-assisted breeding or gene editing.

## Introduction

Apples are one of the most produced and consumed fruits in the world with worldwide production reported at 90 million tonnes in 2016^1^. Widely recognized as a nutritious food source, apples contain significant amounts of polyphenols and other bioactive compounds that contribute to human health and wellbeing. Many polyphenols (e.g., epicatechin, catechin, phloridzin, chlorogenic acid, and proanthocyanins) are strong antioxidants associated with reduced incidence of disease, including cardiovascular disease, metabolic syndrome, and certain cancers^2^. In the US, 22% of the polyphenols in the human diet originate from apples, which makes apples a primary dietary source of these antioxidant compounds^3^. Several epidemiological studies have reported that the consumption of apples can reduce the risk of chronic diseases, including cardiovascular diseases, asthma, various cancers, and type II diabetes^2,4–6^. Thus, apples represent a key source of polyphenols in the human diet that may contribute significantly to disease prevention and overall health.

The concentration of polyphenols in apples varies during ripening and can be influenced by growing conditions^7,8^. For example, phenolic acids and flavonoids in the epicarp and endocarp tissues decrease during ripening^9^. However, it is genetic variability that likely plays the primary role in determining polyphenol concentration because most of the variation in polyphenol concentration is captured by variation among apple cultivars^8,10^. Thus, a major determinant of an apple’s nutritional value is likely determined by its genome, which makes the genetic mapping of polyphenols a promising avenue of scientific inquiry.

The biosynthesis of polyphenols in plants occurs via the secondary metabolism of the phenylpropanoid pathway. This pathway leads to the production of flavonols, flavonoids, anthocyanins, and proanthocyanidins^11^. Biochemical analyses have identified numerous compounds belonging to these polyphenolic groups in apples^12,13^ and genetic mapping studies have revealed genes and enzymes controlling their biosynthesis^14–18^. However, the genetic mapping of apple polyphenols to date has relied exclusively on relatively small (*N* < 170) bi-parental populations^15–17^, which lack the ability to reveal the genetic architecture of polyphenol production across diverse apple germplasm. It therefore remains unknown whether previously identified polyphenol QTL account for variation in diverse apple breeding material, and whether polyphenols are predictable with genome-wide markers using genomic prediction. The nutritional value of crops is increasingly being targeted using genomics-assisted breeding^19,20^, and polyphenol concentration in apple represents a possible target for apple breeders. Thus, to quantify the genetic architecture of apple polyphenols and advance genomics-assisted breeding of apple nutritional content, we conducted genome-wide association studies (GWAS) and genomic prediction using high performance liquid chromatography (HPLC) data of apple extracts in two diverse apple populations.

## Results

### HPLC analyses

The concentrations of six major polyphenolic groups (total phenolics, total hydroxycinnamic acids (HCA), total flavonols, total fluorescence, total anthocyanins, and total phloretin-like compounds) and 14 individual phenolic compounds in two different years are presented in Tables S1 and S2. Out of 19 phenotypes, 17 were significantly correlated between years (Fig. S1). Between-year correlations ranged widely, with the highest between-year correlation for phloridzin (*r* = 0.94, *P* = 4.17 × 10^−34^) and the lowest for total flavonols (*r* = 0.078, *P* = 0.52). In the main text, we report the results from the 2014 data set, while results from 2016 are found in the supplementary material.

**Fig. 1:**
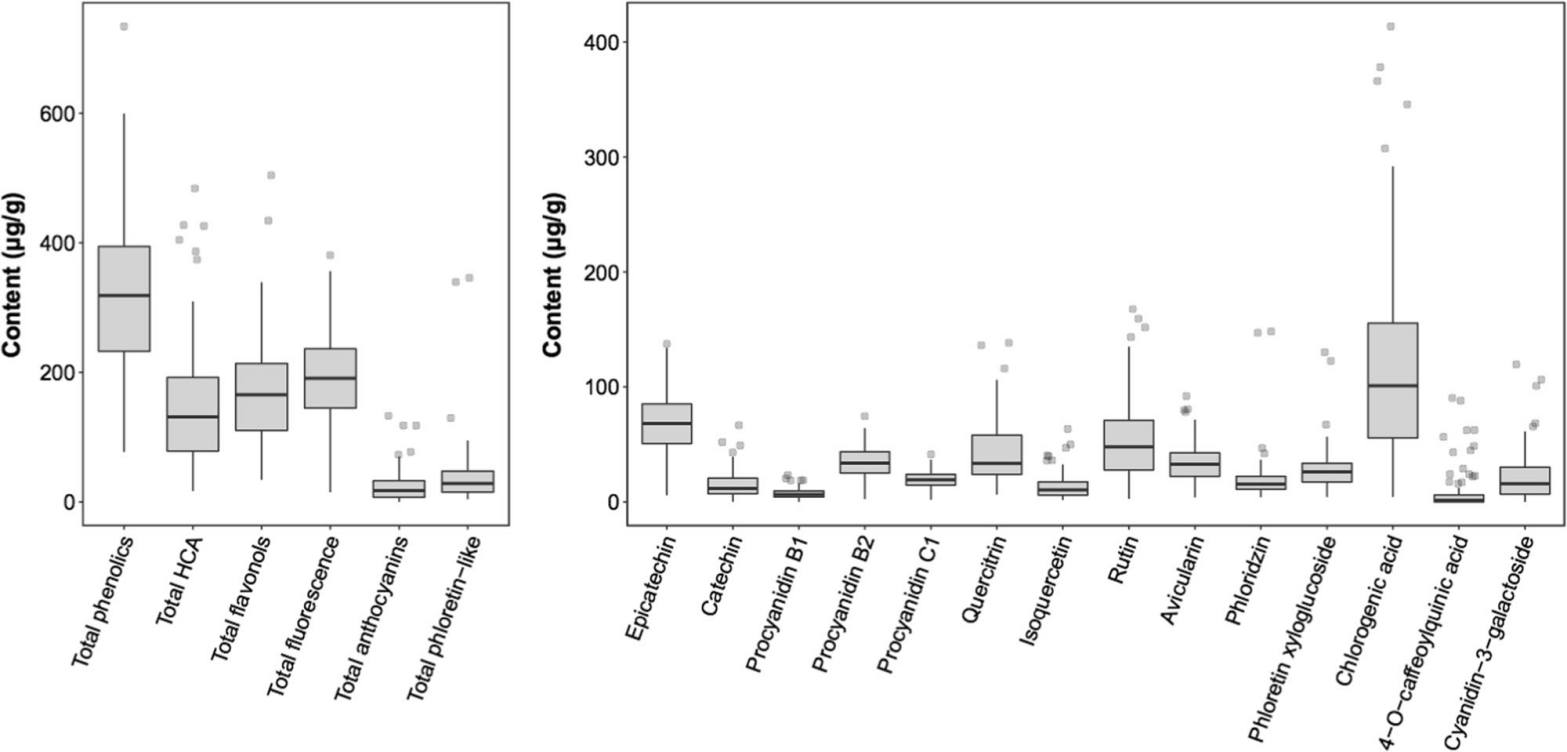
Range and distribution of the concentrations of polyphenols across 136 apple cultivars. The upper and lower hinges of the boxplots correspond to the first and third quartiles, respectively

Polyphenols showed substantial variation among cultivars (Figs. 1 and S2). Major groups of compounds differed by one to two orders of magnitude among cultivars. For example, total phenolic concentration differed by ~10-fold between the cultivar with the lowest (‘Vanda’, 76.88 µg/g) and highest (‘Reinette Russet’, 734.19 µg/g) concentration of total phenolics.

**Fig. 2:**
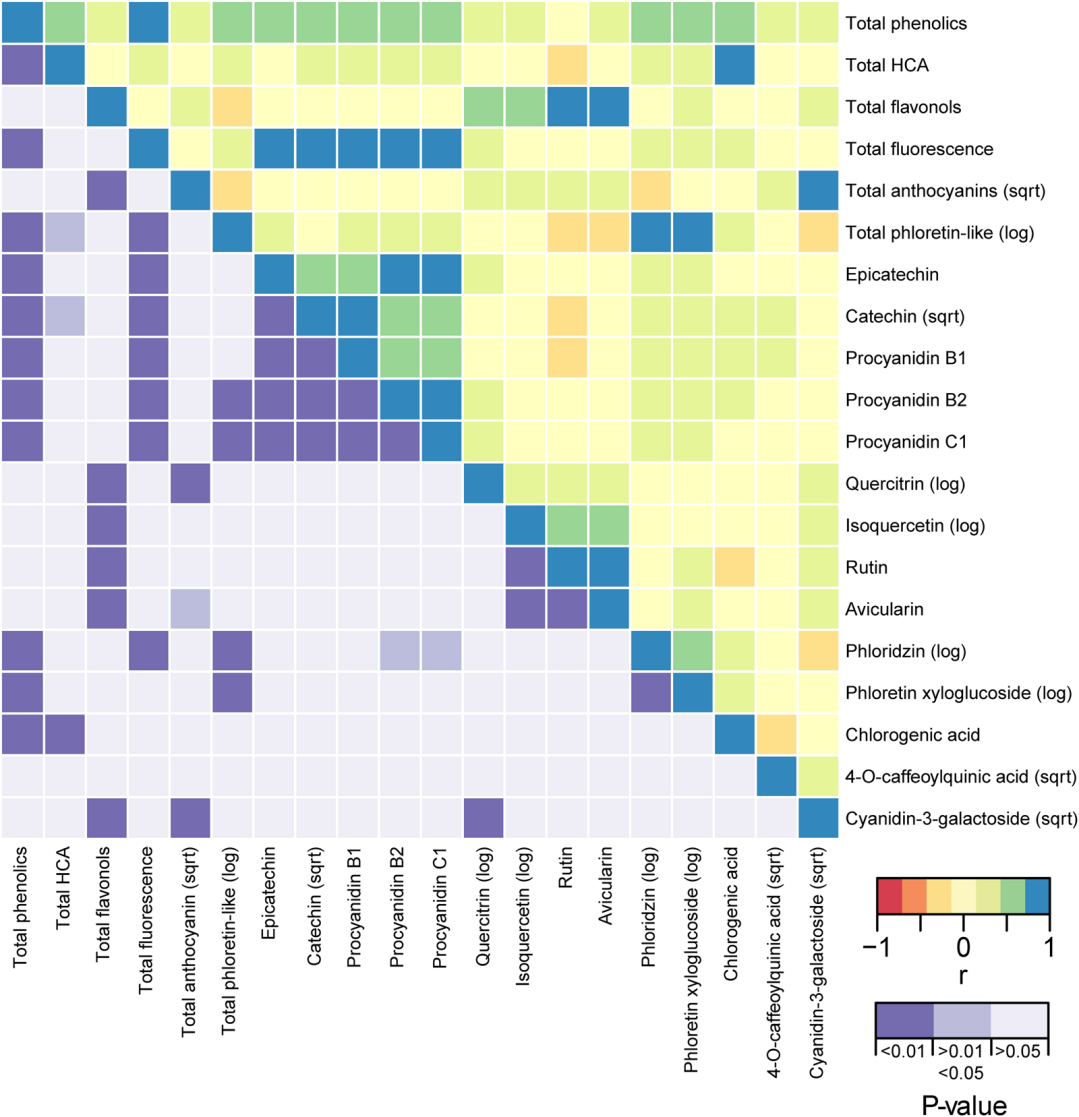
Correlation heat map showing correlations among all pairs of polyphenols measured across 136 apple cultivars. The correlation coefficients (r) are shown above the diagonal. The Bonferonni-corrected *P* values are shown below the diagonal

The strength of the correlations among all pairs of phenotypes are depicted in Fig. 2 and S3. Noteworthy relationships included a strong positive correlation between total phenolics and total fluorescence (*r* = 0.747, *P* = 2.48 × 10^−24^) and significant correlations among epicatechin, catechin, and the three procyanidins (Figs. 2 and S4). Although several pairs of compounds were negatively correlated, none of these were significant after correcting for multiple comparisons. In addition, fruit skin color was positively correlated with total anthocyanin concentration (*R*^2^ = 0.673, *P* < 1 × 10^−15^; Fig. S5) and the degree of fruit browning was positively correlated with total phenolic concentration in fruit (*R*^2^ = 0.299, *P* = 5.91 × 10^−12^; Fig. S6). Finally, scab resistant cultivars had higher concentrations of quercitrin compared to scab susceptible cultivars (W = 3614, *P* = 2.95 × 10^−7^; Fig. S7).

### Genetic mapping and genomic prediction

Significant GWAS results that were replicated in both years are highlighted here in the main text, with figures in the main text showing the results from 2014 because its sample size was larger. All remaining Manhattan plots are found in the supplementary material (Figs. S8 and S9). The position, effect size and list of genes within 100 kb of each significant SNP for both the 2014 and 2016 datasets are found in the supplementary material (Tables S3 and S4).

**Fig. 3:**
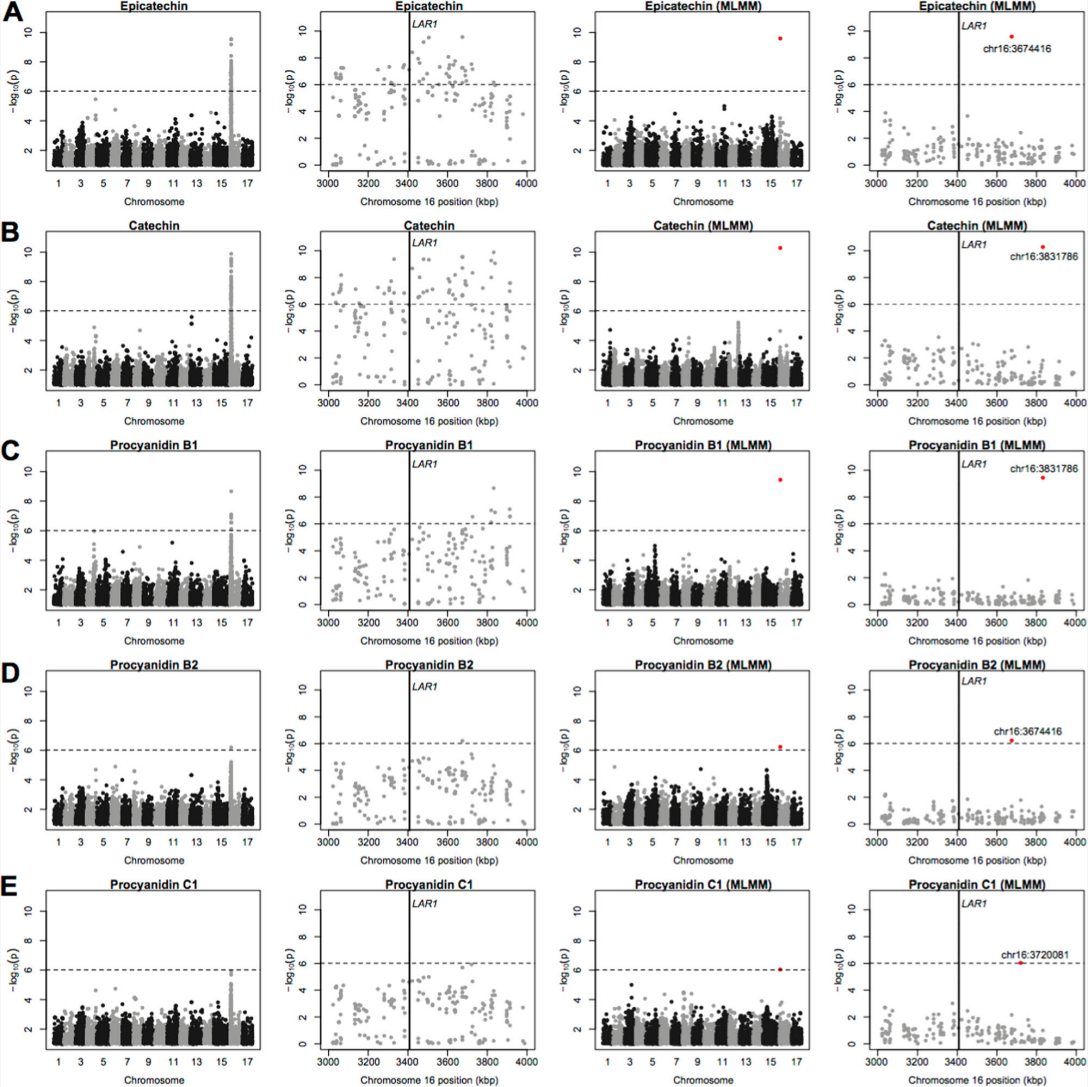
Significant GWAS results for flavan-3-ols and pro-anthocyanidins. Manhattan plots showing the results of GWAS for epicatechin **a**, catechin **b**, Procyanidin B1 **c**, Procyanidin B2 **d**, and Procyanidin C1 **e**. Within each row, the first and second panels show the results of GWAS performed as a series of single-locus tests at the genome-wide and chromosomal scales, respectively. The third and fourth panels show the results of the MLMM GWAS at the genome-wide and chromosomal scales, respectively. A vertical line indicates the location of the LAR1 gene. The red dots are the most significant SNPs identified using MLMM

We found a single, strong association signal on chromosome 16 for the concentrations of catechin, epicatechin, and procyanidins B1, B2 and C1 (Fig. 3). In each case, we found no evidence of allelic heterogeneity, which means the GWAS signal we detected is likely driven by a single variant at this locus. However, the position of the most significant SNP differed slightly between some phenotypes (Fig. 3; Tables S3 and S4). A single candidate gene was identified within this genomic region: leucoanthocyanidin reductase (*LAR1*). The same locus on chromosome 16 was also detected in the GWAS for total fluorescence, which largely captured the sum of the concentrations of these five compounds together with unidentified compounds of similar chemical structure (Figs. S8 and S9).

**Fig. 4:**
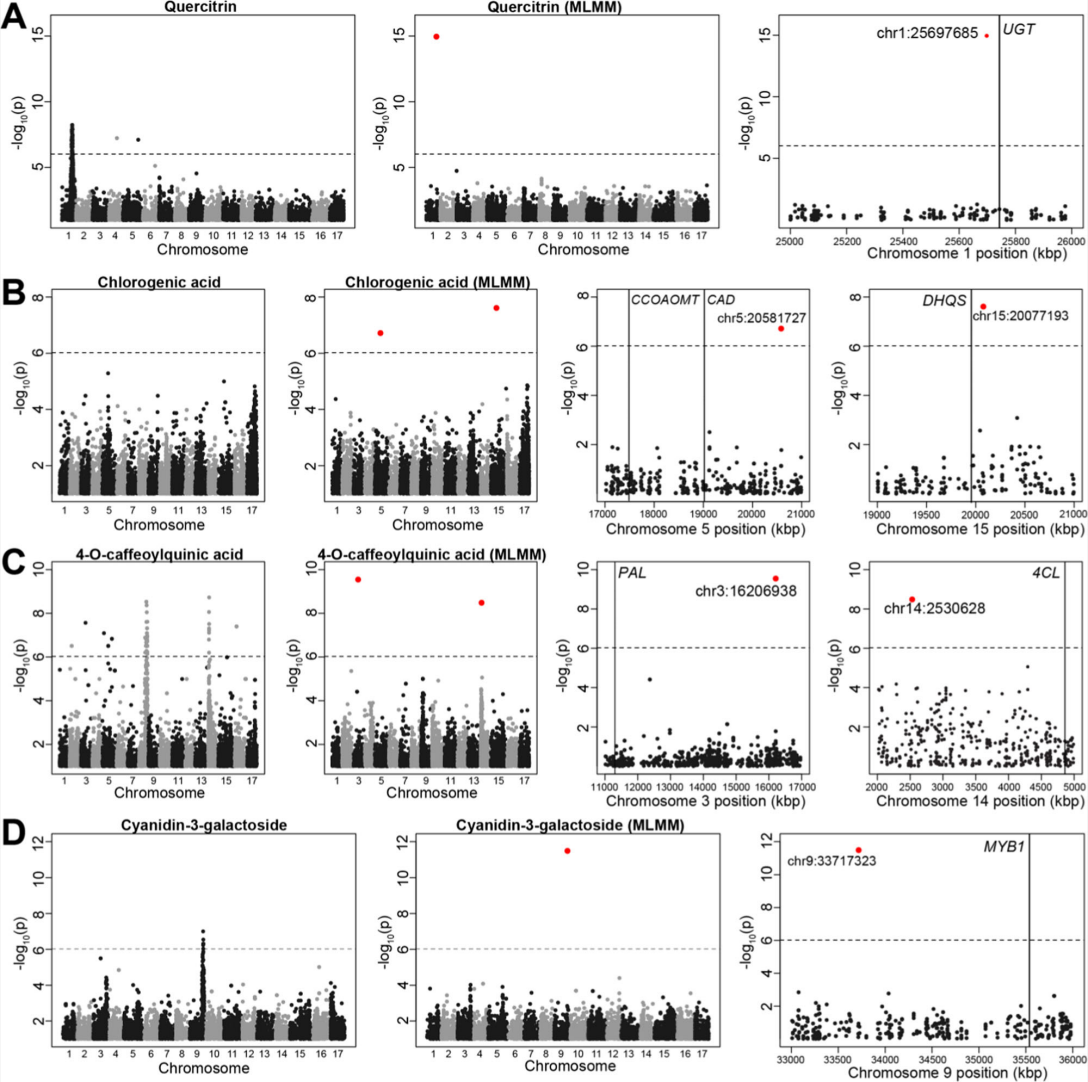
Significant GWAS results for several polyphenols. Manhattan plots showing the results of GWAS for quercitrin **a**, chlorogenic acid **b**, 4-O-caffeoylquinic acid **c**, and cyanidin-3-galactoside **d**. Within each row, the first panel shows the results of GWAS performed as a series of single-locus tests. The subsequent panels show the results of the MLMM GWAS at the genome-wide and chromosomal scales. A vertical line indicates the location of candidate genes. The red dots are the most significant SNPs identified using MLMM

Significant genotype-phenotype associations were also detected for quercitrin, chlorogenic acid, 4-*O*-caffeoylquinic acid, and cyanidin-3-galactoside (Fig. 4). The GWAS hit on chromosome 1 for quercitrin (chr1:25697685) occured 94 kb upstream of a UDP-glycosyltransferase gene (*UGT*; Fig. 4a). Two significant associations were detected for chlorogenic acid. The first (chr5:20581727) was within 3 Mb of two candidate genes: a caffeoyl-CoA O-methyltransferase gene (*CCOAOMT*) and a cinnamyl alcohol dehydrogenase gene (*CAD*). The other SNP significantly associated with chlorogenic acid (chr15:20077193) was found just slightly more than 100 kb downstream of a 3-dehydroquinate synthase gene (*DHQS;* Fig. 4b). GWAS for 4-*O*-caffeoylquinic acid also produced two significant associations. The first (chr3:16206938) was found 5 Mbp from a phenylalanine ammonia-lyase gene (*PAL*). The second GWAS hit for 4-*O*-caffeoylquinic acid (chr14:2530628) was ~2.3Mbp upstream of a 4-coumarate-CoA ligase-like gene (*4* *CLL*; Fig. 4c). Finally, the hit for cyanidin-3-galactoside on chromosome 9 (chr9:33717323) was 1.8 Mb from the *MYB1* transcription factor (MD09G1278600) that regulates apple skin color^21,22^ (Fig. 4d).

When performing GWAS for each of the six major polyphenolic groups, we often discovered the same association as we did for the individual compounds within the group. For example, SNPs significantly associated with total HCA, total fluorescence, and total anthocyanins were the same as those for chlorogenic acid, epicatechin/catechin/procyanidins, and cyanidin-3-galactoside, respectively (Figs. S8 and S9).

**Fig. 5:**
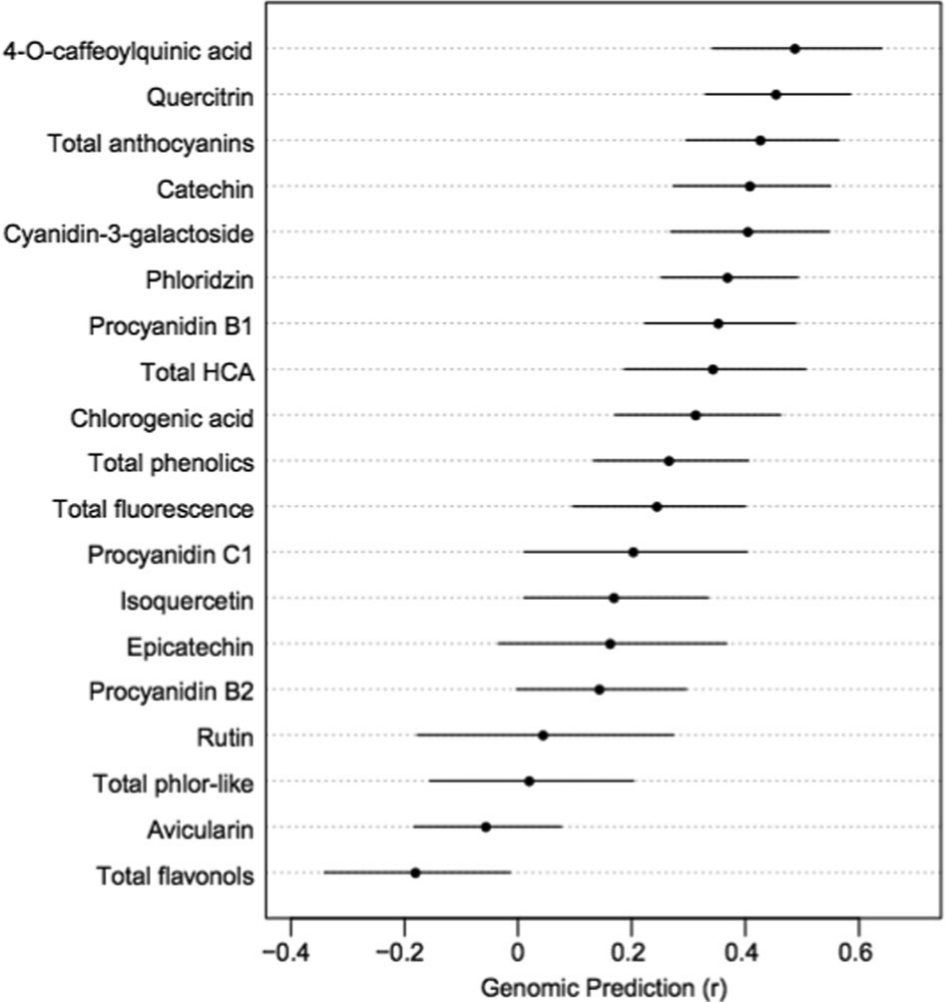
Estimates and standard deviations of genomic prediction (r) values for all polyphenols measured across 136 apple cultivars

Finally, the genomic prediction accuracies (r) varied widely from −0.18 for total flavonols to 0.49 for 4-*O*-caffeoylquinic acid (Figs. 5 and S10) and were positively correlated between years (R^2^ = 0.373, *P* = 0.007; Fig. S11).

## Discussion

The concentrations of polyphenols observed in the present study were in line with previous values measured across <20 cultivars^8,23,24^, however our larger sample size of 136 cultivars resulted in a far greater range of values with up to a 10-fold difference in concentration of some polyphenols across cultivars (Fig. 1). For example, chlorogenic acid is a primary source of antioxidants in apples and concentrations in the present study varied from 4.25 to 413.6 µg/g. The observed wide variation in polyphenol concentration suggests that eating apples may provide varying health benefits depending on which cultivar is consumed.

Two russetted accessions, ‘Reinette Russet’ and ‘SJCXN362′, were outliers with respect to phloridzin and phloretin xyloglucoside concentration. Most cultivars had very low concentrations of these compounds, but these two accessions contained approximately twice the concentration of phloretin xyloglucoside and three times the concentration of phloridzin compared to the accession with the next highest values (Tables S1 and S2). Phloridzin is the most prominent dihydrochalcone in apples^25^ and has anti-diabetic, anti-cancer and anti-inflammatory effects^26–28^. These observations suggest that selection against russetting in an apple breeding program may result in a reduction in nutritional quality.

The strong positive correlations among catechin, epicatechin, and the procyanidins B1, B2, and C1 support the notion that the molecular control of all these compounds is regulated by a common mechanism^17^ (Figs. 2 and S4). Although we observed no significant negative correlations among phenotypes, the negative relationship between rutin and catechin suggests they may compete for the same precursor.

The strong correlation between red coloration of apple skin anthocyanin concentration (Fig. S5) is consistent with the well-known relationship between color and anthocyanins in apples^29^. Previous work has shown that about half of the polyphenols in apples are in the skin, while the other half are found in the flesh^8^. In another study, the skin showed a 2–9 times higher phenolic concentration than the pulp^30^. This suggests that selection for apple skin characteristics may result in a larger effect on the nutritional value of an apple than selection for features of the pulp.

Previous work has shown that the degree of enzymatic browning is correlated with polyphenol concentration in apples^31^. Our observed correlation between browning and total phenolic concentration supports the notion that polyphenol concentration is a useful proxy for enzymatic browning potential (Fig. S6). Because enzymatic browning is a major problem for the fruit processing industry, apples with low polyphenol concentration, and thus potentially lower nutritional value, will likely continue to be most attractive for this industry^32^.

Scab resistant cultivars had higher concentrations of quercitrin compared to scab susceptible cultivars (Fig. S7). The natural defensive reactions of apples against various diseases, especially apple scab (*Venturia inaequalis*), include the production of polyphenols. Numerous polyphenols have been found at higher concentrations in scab resistant cultivars when compared to scab susceptible cultivars^30^. In particular, total flavanol concentration in the skin of scab resistant cultivars was found to be 3-fold higher than in susceptible cultivars^33^. Thus, breeders focussing on scab resistance as a breeding target may simultaneously be enhancing the nutritional value of breeding material by selecting for higher concentrations of polyphenols.

The collection of phenotype data in two separate years from replicated trees across two orchards in the present study provided insight into potential sources of variation in polyphenolic concentration. Although most phenotypes showed strong and significant correlations between years, 4 of the 19 phenotypes had *r* < 0.5 and were thus strongly affected by either differences in location, year or sources of phenotyping noise (Fig. S1). Similarly, we found that some phenotypes had highly variable prediction accuracies between years, and thus appeared more highly heritable in one year than in the other. For example, total HCA had a genomic prediction accuracy of 0.35 in 2014, and a value of 0.059 in 2016 (Fig. S11). We focused on genetic mapping results that were replicated in both years, however, several phenotypes that showed significant genotype-phenotype associations in only a single year (e.g., total flavonols, rutin, and phloridizin; Figs. S8 and S9) may be worthy of further investigation.

A clear trend was observed in the GWAS results for the flavan-3-ols and pro-anthocyanidins, with a large peak on chromosome 16 for epicatechin, catechin, procyanidin B1, B2, and C1 (Fig. 3). As flavan-3-ols form the building blocks of proanthocyanidins, a single GWAS signal was expected given the strong correlations among all five of these phenotypes (*R* = 0.51–0.98; Fig. S4). Among these correlated phenotypes, the location of the most significant SNP differed slightly in some cases, accounting for up to 50% of the phenotypic variance. This region on chromosome 16 was previously highlighted as a QTL hotspot for epicatechin, catechin, and proanthocyanidins based on linkage mapping in bi-parental populations^15,17^. In the present study, we found slight differences in the location of the most significant SNP across phenotypes, but all were within the boundaries of the QTL hotspot previously reported. Leucoanthocyanidin reductase (*LAR1*) has been identified as a putative candidate gene for this hotspot, as it is thought to catalyze the conversion of leucocyanidin to catechin. However, this region also contains several transcription factors of different classes (e.g., *MYB*, *bHLH*, *bZIP*, *AP2*), which could also be affecting phenolic levels (Tables S3 and S4). Khan et al.^18^ examined expression profiles of several genes within this genomic region during different stages of fruit development and found that only *LAR1* showed a significant correlation between transcript abundance and metabolite content. Chagne et al.^15^ proposed that the causal mutation driving this signal on chromosome 16 is in the promoter region of *LAR1*, in a site recognized by the transcription factors regulating it, and that it does not result in a complete loss of function of *LAR1*. This hypothesis is consistent with our finding of a large effect locus in or around *LAR1*, and our intention is to conduct GWAS with more samples and more markers in the future in the hope that these provide sufficient mapping resolution to identify putatively causal variants.

GWAS peaks for several other phenolic compounds were also observed including for the flavonol, quercitrin, on chromosome 1 (Fig. 4a). Other studies have found hits for flavonols on chromosome 1 and suggested that a uridine diphosphate-dependent glycosyltransferase gene (*UGT*) and/or a flavonoid 3′-hydroxylase (*F3*′*H*) gene as potential candidate genes underlying this signal^16,17^. In the present study, the most strongly associated SNP with quercitrin was found approximately 44 kb upstream of a *UGT* gene (MD01G1148700). Plants contain large families of *UGT*s, and there are dozens or perhaps even hundreds of *UGT* genes in apples^34,35^. *UGT*s mediate the glycosylation of flavonoids, and quercitrin is produced by the glycosylation of the flavonoid quercetin. The glycosylation of secondary metabolites increases the solubility and stabilization of flavonoid compounds^36^, and specific *UGT*s have been identified that glycosylate flavonoids into potent antioxidants like phloridzin^34,35^. To the best of our knowledge, however, no specific *UGT* has been associated with the formation of quercitrin in apples. We hypothesize that the GWAS signal we observed here on chromosome 1 is the result of variation in a specific *UGT* gene (MD01G1148700) that regulates the glycosylation of quercetin and thus the concentration of quercitrin. To further investigate the function of this *UGT* gene, we plan to determine whether it in fact uses quercetin as a substrate, and whether the expression of this gene correlates with quercitrin concentration across diverse apple cultivars. Ultimately, markers at this locus could be leveraged for marker-assisted breeding, or the antioxidant content of novel cultivars may be mediated by introducing variation at this locus via genome editing.

The GWAS for chlorogenic acid produced two significant hits on chromosomes 5 and 15, suggesting that variation at two independent loci affect this trait (Fig. 4b). We identified three promising candidate genes at these loci including caffeoyl-CoA *O*-methyltransferase (*CCOAMT*; MD05G1083900), cinnamyl alcohol dehydrogenase (*CAD*; MD05G1089900), and 3-dehydroquinate synthase (*DHQS*; MD15G1242600). Both CCOAMT and CAD are enzymes associated with the biosynthesis of hydroxycinnamic acids through the phenylpropanoid pathway, which also supplies intermediates for the synthesis of phytoalexins, flavonoids and tannins^37^. Although not directly involved in the final step of chlorogenic acid biosynthesis, CCOAMT is active upstream of its production through the conversion of caffeoyl-CoA to feruloyl-CoA^38^ and has been associated with chlorogenic acid accumulation in coffee^39^. CAD converts cinnamyl alcohol to cinnamaldehyde. A CAD gene was found to be strongly expressed in ripening receptacle tissue in strawberries^40^, but, to our knowledge, no CAD gene has yet been characterized in apples. Finally, DHQS is involved in the shikimate pathway by catalyzing key substrates for chlorogenic acid biosynthesis^41^. All three of these genes represent candidates worthy of future investigation given their previously established relationships to chlorogenic acid production.

Previous linkage mapping studies in bi-parental apple populations found strong associations with chlorogenic acid on chromosome 17^15,17^, and have suggested shikimate/quinate O-hydroxycinnamoyl transferase (HCT/HQT) genes as potential candidates. While there are no SNPs on chromosome 17 significantly associated with chlorogenic acid in the present study, there was a suggestive GWAS signal on chromosome 17 for chlorogenic acid (Fig. 4b). Indeed, a HCT/HQT gene (MD17G122510) is located directly within the suggestive peak on chromosome 17 (Fig. S12). Thus, we conclude that the peak on chromosome 17 likely represents a true signal of association driven by variation in or around HCT/HQT, and that this signal was not significant in the current study due to the low SNP density, noisy phenotype data, and/or other factors that reduced the power of our GWAS. Our failure to detect this association leads us to conclude that other suggestive but non-significant GWAS signals we observed for other traits may also represent true genotype-phenotype associations.

Another hydroxycinnamic compound, 4-*O*-caffeoylquinic acid, produced significant GWAS hits on chromosomes 3 and 14 (Fig. 4c). The first panel of Fig. 4c shows a Manhattan plot that does not account for multiple loci, and a reasonable interpretation of this plot would suggest two independent loci controlling this phenotype on chromosomes 8 and 14. However, the subsequent panels reveal that the signal on chromosome 8 disappeared when conditioning on the top hit on chromosome 14. We reason that this is most likely due to genome mis-assembly: the SNPs significantly associated with this phenotype on chromosomes 8 and 14 are in fact physically close and in strong LD with each other despite appearing as independent genomic regions in the genome assembly. Thus, the use of the MLMM is not only helpful in determining the genetic architecture of a trait (i.e., the number of independent loci involved), but can also help clarify genome assembly issues.

A stable signal of association for 4-*O*-caffeoylquinic acid and its precursor, 4-*p*-coumaroylquinic acid, has been detected by three previous genetic mapping studies^15–17^. Verdu et al.^16^ proposed flavonoid 3′-hydroxylase (*F3*′*H*) or flavonoid 3′,5′-hydroxylase (*F3*′*5*′*H*) as potential candidate genes for the signal they discovered on chromosome 14 for hydroxycinnamic acids, but neither *F3*′*H* nor *F3*′*5*′*H* genes were found within the interval we identified on chromosome 14. Although nearly 2.5 Mb from the most significant SNP, our candidate gene for the signal on chromosome 14 was 4-coumarate-CoA ligase-like (*4CLL*), which converts 4-courmarate into 4-courmaroyl CoA. Interestingly, 4-courmaroyl CoA is a precursor of *p*-coumaroylquinic acid, which shares QTL intervals with 4-*O*-caffeoylquinic acid according to previous work^15,16^.

The conversion of phenylalanine to *p*-coumaroyl-CoA, with cinnamic acid and *p*-coumaric acid acting as intermediates, is catalyzed sequentially by phenylalanine ammonia lyase (PAL), cinnamate 4-hydroxylase (C4H) and 4-cinnamoyl-CoA ligase (4CL). Although nearly 5 Mb from our association signal for 4-*O*-caffeoylquinic acid on chromosome 3 (Fig. 4c), phenylalanine ammonia-lyase (*PAL*; MD03G1121500) may be a candidate gene for the signal we observed here. PAL is an enzyme that catalyzes the production of cinnamic acid, a precursor to the hydroxycinnamic compounds. Because PAL is the starting enzyme of the phenylpropanoid pathway, it plays a crucial role in controlling the biosynthesis of acyl-quinic acids^38^. The rapid decay of LD we observed in apple GWAS populations highly similar to the one studied here^42–44^ suggests it is unlikely that causal alleles will be found megabases away from the association signals detected in the present study. However, we propose these candidates despite this observation because we were unable to accurately quantify the physical distance over which association signals potentially span in this population. With denser marker data and larger sample sizes, a more accurate delimiting of the physical intervals will be achievable in future apple GWAS.

A strong GWAS peak was found for cyanidin-3-galactoside on chromosome 9 (Fig. 4d), and the most strongly associated SNP at this locus was also the most significantly associated SNP with total anthocyanins (Fig. S8). These associations were expected because cyanidin-3-galactoside is the most prominent anthocyanin in apples^13^ and QTL for apple skin color repeatedly co-locate to this genomic region^21,22,42,45–49^. A SNP (ss475879531; chr9:33001375) used to predict skin color by apple breeders^48^ is located 666 kb upstream from the most significant SNP we identified. However, a recent study identified a retrotransposon insertion 1 kb upstream of the *MYB1* gene (chr9:35,541,127–35,541,721) that likely causes the red-skinned phenotype^49^. Although this putatively causal allele was 1.8 Mb downstream from our top GWAS hit, it overlapped with the broad GWAS peak we observed for both cyanidin-3-galactoside and total anthocyanins (Fig. S13). If apple breeders have been selecting for red-skinned apples, our broad GWAS signal in this region may be due to elevated levels of linkage disequilibrium (LD) caused by the action of positive selection for the red-skinned phenotype. A more powerful GWAS with more samples and markers could determine whether the signal we observed here co-locates with the recently identified putatively causal retrotransposon at the *MYB1* gene.

Finally, genomic prediction accuracies for polyphenolic concentrations ranged from below 0 (not predictable) to 0.49 (Fig. 5). Using the same population studied here, we previously found prediction accuracies ranged from 0.08 for change in firmness during storage to 0.72 for scab resistance^44^. The prediction accuracies from our diverse population are expected to be lower than those observed in apple breeding populations in which relatedness is higher between the training population and the population in which prediction takes place. For example, a cross-validation procedure for six fruit quality traits produced accuracies ranging from 0.67 to 0.89 in a New Zealand apple breeding population derived from 4 female and 2 male parents^50^. In another study, from a training population of 20 full-sib families, prediction accuracies reached a maximum of 0.5 across 10 traits in trees with high degrees of relatedness to the training population^51^. If we assume that prediction accuracies >0.2 indicate that a trait responds well to improvement via genomic selection^51^, then more than half (12/19) of the traits studied here have potential for improvement via genomic selection.

Overall, our results indicate that the concentrations of polyphenols vary dramatically in a diverse apple population and that much of this variation is heritable and predictable using genetic markers. In cases where we discovered significant genotype-phenotype associations using GWAS, the proportion of the phenotypic variance (R^2^) explained by the top SNPs ranged from 0.31 to 0.63 (Table S2). Previous GWAS in apple identified SNPs accounting for up to 33% of the variance in flowering time^52^ and 25% of the variance in fruit quality traits^53^. Our relatively high effect size estimates may be partially due to our small sample sizes. Even taking our sample size into account, our observations suggest that the expression of several apple polyphenols is under relatively simple genetic control, and that the markers we have identified are in strong LD with causal genetic variation underlying polyphenol expression. We corroborated several previously detected associations for epicatechin, catechin, procyanidin B1, B2, C1, and anthocyanins, and discovered novel loci and potential candidate genes for chlorogenic acid, quercitrin and 4-*O*-caffeoylquinic acid. Several of the SNPs reported here are strong candidates for use in marker-assisted breeding. For the polyphenols without significant GWAS results, we demonstrated that they are often predictable using genome-wide SNPs and thus may be amenable to breeding using genomic selection. Despite their importance in the human diet, polyphenols are not widely targeted by apple breeders to our knowledge. Our results suggest that genomics-assisted breeding for enhanced polyphenols could be fruitful and lead to novel cultivars with enhanced nutritional properties.

## Materials and methods

### Apple germplasm

Apple cultivars used for this study originated from the Nova Scotia Fruit Growers’ Association (NSFGA) Cultivar Evaluation Trial (CET) based at Agriculture and Agri-Food Canada’s (AAFC) Kentville Research and Development Centre in Nova Scotia, Canada as described previously^44^. Phenotype data were collected from fully mature trees from the CET in 2014, and we refer to this data set as the “2014 data”. Fruit skin color was scored by eye as the percentage of red blush covering the fruit surface, and flesh browning was assessed on fruit cut longitudinally and exposed to air for 40 min, followed by scoring on a scale from 1 (no browning) to 6 (severe flesh browning). Scab resistance/susceptibility was scored as a binary trait according to information obtained from breeders upon introduction of cultivars into the orchard.

Following measurements taken at harvest, the remaining fruit were kept at 0.8–1 °C in stacked, perforated plastic bins covered in plastic sheets. After 1 month of storage, fruit were removed for phenolic analyses. The distributions of phenolic phenotypes were visualized using the “geom_boxplot” function in the ggplot2 R package^54^. Next, these phenotypes were correlated with fruit skin color using Pearson’s product-moment correlation test, flesh browning using Spearman’s rank correlation test, and scab resistance presence using the Wilcoxon rank sum test. *P* values are reported after Bonferonni correction for multiple comparisons for all of these comparisons. All statistical analyses were performed in R^55^.

Of the 136 cultivars evaluated in 2014, 85 were evaluated again in 2016. The 2016 phenotype data were collected from 85 cultivars from the CET that were grafted onto M.9 rootstock in the spring of 2012 and planted in an adjacent orchard in the spring of 2013. We refer to this dataset as the “2016 data”. Cultivars were planted in two different randomized blocks with a single tree in each block. The orchard was maintained to industry standards and no supplemental irrigation applied. Trees were hand thinned in mid-July to adjust crop load to commercial standards of one fruit per cluster, with 10 to 15 cm between each fruit. Fruit were harvested based on maturity assessment using a starch-iodine or starch-to-sugar conversion test, seed color, background fruit skin color, and presence of fruit drop. Following harvest, fruit were kept at 0.8–1 °C in stacked, perforated plastic bins covered in plastic sheets to retain moisture. After 1 month of storage, fruit were removed for phenolic analyses. Apple tissue with peel and flesh was frozen and ground in liquid nitrogen, and stored at −86 °C until extraction. There were 70 common cultivars phenotyped in 2014 and 2016, and we calculated the Pearson correlation between years for each phenotype using the cor.test function in R.

### Phenolic analysis using HPLC

High performance liquid chromatography (HPLC) was used to analyze the phenolic compounds in apple tissue that included both peel and flesh^56^. Briefly, 0.5 g of ground tissue was extracted twice with 0.7 mL of extraction solvent (80:20 methanol: water, V/V, 0.1% formic acid) in micro-centrifuge tubes. The samples were mixed for 10 s, sonicated for 20 min before mixing for another 10 s, followed with centrifugation at 10,000 × *g* for 10 min at room temperature to pellet suspended tissue (Thermal ICE Microlite). The supernatants from the two extractions were pooled and transferred to weighed microcentrifuge tubes and dried in a vacuum centrifuge (Thermo Fisher) for 16 h, to determine extract yield. The dried extracts were re-dissolved in 1 mL 10% methanol, 0.1% formic acid and mixed via sonicating for 10–15 s then vortexing for 10 s. Extracts were centrifuged at 10,000 × *g* for 10 min at room temperature and supernatants were transferred to HPLC vials for injection. Meanwhile, the percent dry weight of the tissue samples was also determined at the same time that extractions were conducted, on separate duplicate ~1 g aliquots of the ground tissue, using a vacuum drying oven heated to 100 °C for 24 h.

A HPLC system with photo diode array detector (PDA) and fluorescence detector (Waters, Milford, MA) was used to quantify polyphenols and anthocyanins in the apple extracts (flesh with peel) with a few modifications. Liquid chromatographic separation was achieved using an Agilent Poroshell 120 SB C18 2.7 µ 3.0 × 75 mm column (Agilent, Palo Alto, CA) at room temperature with a flow rate of 0.5 mL/min. The mobile phase consisted of 0.8% trifluoroacetic acid in water (solvent A) and 0.68 % trifluoroacetic acid in acetonitrile (solvent B) with a solvent elution gradient as follows: 0 min: 2% B, 2 min: 2% B, 22 min: 6% B, 30 min: 12% B, 60 min: 35% B, 62 min: 100% B, 64 min: 100% B, 65 min: 2% B, re-equilibrating 10 min before next injection. Injection volume was 30 µL, and detection was 200–600 nm on PDA, extracting chromatograms at 280 nm (total phenolics), 320 nm (total hydroxycinnamates (HCA)), 360 nm (total flavonols) and 520 nm (total anthocyanins). We also quantified total fluorescence with 228 nm as excitation and 324 nm as emission, which individually detects catechin, epicatechin, procyanidin B1, procyanidin B2 and procyanidin C1. For phloretin-like compounds, we detected and extracted from the UV profiles at 280 nm for the 2014 data because numerous cultivars showed extra phloretin-like peaks not fully captured by the specific compounds quantified using standards listed below. For the 2016 data, however, only three cultivars (‘Coop29′ (11.79 µg/g), ‘Reinette Russet’ (46.16 µg/g) and ‘Britegold’ (4.66 µg/g)) showed other phloretin-related peaks and therefore no results for “total phloretin-like” are shown in Fig. S1 for the 2016 data. Retention times and UV/Vis profiles were compared to pure standards to identify peaks and quantify specific compounds. Catechin, epicatechin, chlorogenic acid (5-*O*-caffeoylquinic acid), 4-*O*-caffeoylquinic acid, phloridzin, quercetin, quercitrin, rutin, isoquercetin, cyanidin-3-glucoside were purchased from Sigma-Aldrich Canada Co. (Oakville, Ontario) and used as standards. For phloretin xyloglucoside, the phloridzin standard was used. Standards for procyanidin B1, procyanidin B2, procyanidin C1 and avicularin were purchased from the Indofine Chemical Co. (Hillsborough, NJ). Standards were used to calibrate the HPLC under the same conditions (10% methanol, 30 µL injection) to quantify the respective phenolic compounds in the extracts. Two individual extractions were conducted and results were averaged from the replicates.

### Genotype Calling

Genotype data for the CET were generated via genotyping-by-sequencing (GBS)^57^ as described previously^44^ except that reads were aligned to the most recent reference genome version GDDH13 Whole Genome v1.1^58^. Raw VCF files were filtered using VCFtools^59^ to include only bi-allelic SNPs with a minor allele frequency (MAF) >1%. The two VCF files were merged using a custom perl script that preferentially kept SNPs generated from the *Pst*I-*Eco*T22I file because these SNPs tended to have higher coverage. SNPs were imputed using LinkImputeR^60^, allowing for PositionMiss(0.7), SampleMiss(0.7), and Depth(6), which resulted in 154,153 SNPs with an imputation accuracy of 0.956. Finally, two final genotype tables were produced, one for the samples phenotyped in 2014 (i.e., the 2014 data), and one for the samples phenotyped in 2016 (i.e., the 2016 data), by removing SNPs within each set of samples that had heterozygosity >90% and MAF < 5% using PLINK^61^ separately for the 2014 and 2016 data. This resulted in 98,584 SNPs across 136 samples for the 2014 data, and 97,886 SNPs across 85 samples for the 2016 data. The population genetic structure of these samples was previously described in^44^.

### GWAS and genomic prediction

GWAS was conducted using the multi-locus mixed model (MLMM) R package^62^ controlling for both population structure (Q) using principal components (PCs) and relatedness using a kinship matrix (K). If the Shapiro-Wilk test for normality produced a value <0.91 for any given phenotype, the phenotype data were transformed to improve normality using either the log or square-root transformation. The MLMM is a modified mixed linear model that uses stepwise regression to incorporate significant SNP markers as cofactors. For this study, the optimal MLMM model for each phenotype was selected using the extended Bayesian information criterion (EBIC). The percentage of variance explained by the SNPs included in the selected model was determined from the partitioning of phenotypic variance for each forward inclusion and backward elimination of the model. The significance threshold for MLMM was determined separately for the 2014 and 2016 data, using the “simpleM” package in R^63^, which calculates the effective number of independent tests (M_*eff*_). The significance threshold was drawn as -log_10_(α/M_*eff*_) where α was set to 0.05 (dashed line in GWAS plots). If a SNP crossed the M_*eff*_ threshold and was deemed significantly associated with a phenotype, a 100 kbp window centered on that SNP was explored for candidate genes using the Genome Database for Rosaceae (GDR)^64^. Keyword searches for genes on chromosomes with significant GWAS results were also completed using GDDH13 Whole Genome v1.1 (https://www.rosaceae.org/search/genes).

Genomic prediction was performed using the “x.val” function in the R package PopVar^65^. The rrBLUP model was selected and 5-fold (nFold = 5) cross-validation was repeated 3 times (nFold.reps = 3) using the same SNP sets used for GWAS. All other default parameters were used. The correlation of the genomic prediction accuracy (r) between the 2014 and 2016 data sets was calculated using a Pearson’s correlation.

## Data availability

All phenotype data are available in the Supplementary material. The genotype data are available from the Dryad Digital Repository: 10.5061/dryad.8fb46m5.

## Supplementary information

Supplementary materials

Supplementary Table 1

Supplementary Table 2

Supplementary Table 3

Supplementary Table 4
