# Supplementary material for: Genome-wide association studies in apple reveal loci of large effect controlling apple polyphenols
Source: Hortic Res. 2019 Sep 7;6:107. doi: 10.1038/s41438-019-0190-y (PMC6804656; doi:10.1038/s41438-019-0190-y)

**Supplementary Tables and Figures**

**Supplementary Tables are available at** http://www.cultivatingdiversity.org/supptables.html

**Supplementary Table 1:** Phenotype data from 2014.

**Supplementary Table 2**: Phenotype data from 2016.

**Supplementary Table 3**: List of significant SNPs for the GWAS of the 2014 phenotype data. Only SNPs identified as significantly associated with a trait according to the MLMM GWAS are shown. The genomic coordinates, P value, R squared and candidate genes within 100kb of the SNP are shown.

**Supplementary Table 4**: List of significant SNPs for the GWAS of the 2016 phenotype data. Only SNPs identified as significantly associated with a trait according to the MLMM GWAS are shown. The genomic coordinates, P value, R squared and candidate genes within 100kb of the SNP are shown.

**Figure S1**: Between-year phenotype correlations. Distribution of R^2^ values for the correlation between the two sets of phenotype data from 2014 and 2016. Significant correlations are marked with an asterisk.


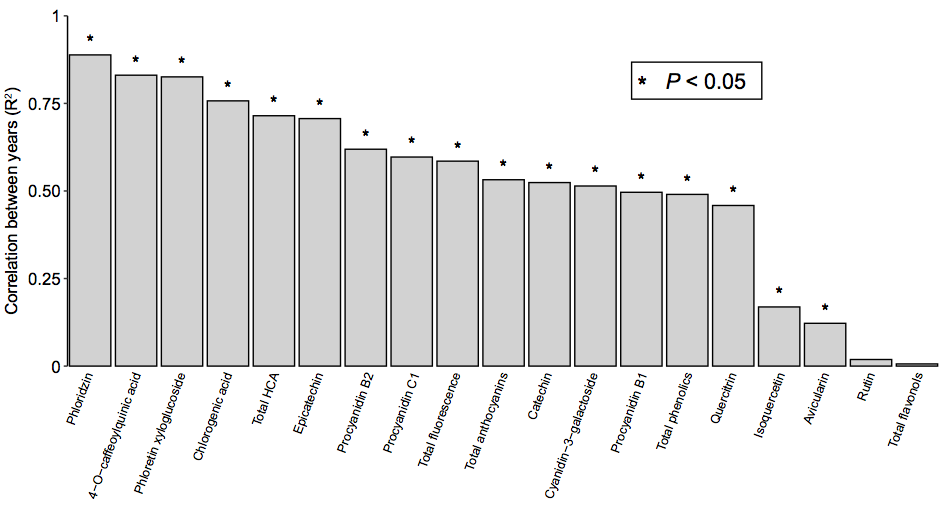


**Figure S2**: Range and distribution of the concentrations of polyphenols for the 2016 data.


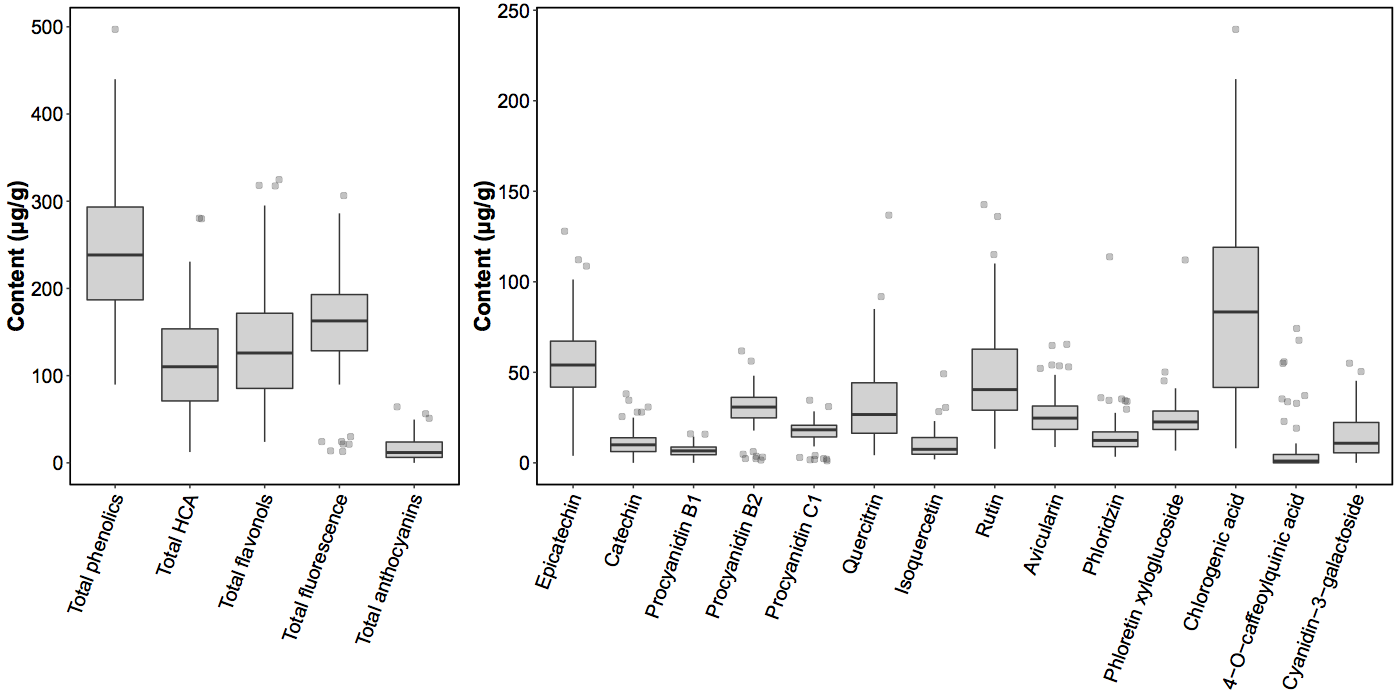


**Figure S3**: Correlation heat map showing correlations among all pairs of polyphenols from the 2016 data. The correlation coefficients (r) are shown above the diagonal. The Bonferonni-corrected P values are shown below the diagonal.
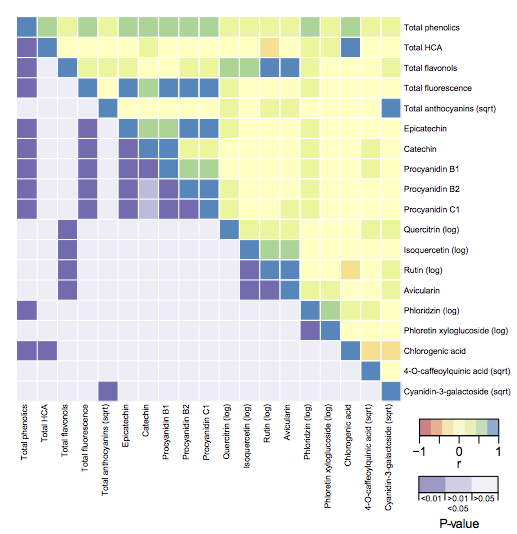


**Figure S4**: Strong correlations among catechin, epicatechin and the procyanidins from the 2014 data. Below the diagonal, dot plots display the relationship between two phenotypes. Above the diagonal, the correlation coefficients are shown. And along the diagonal, a density distribution of each phenotype is shown.
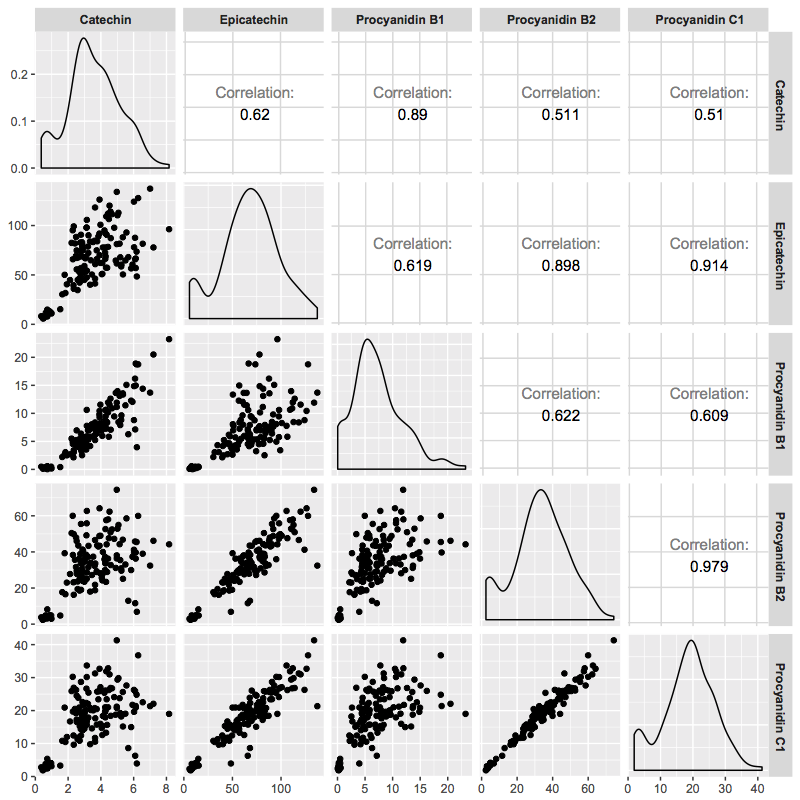


**Figure S5**: The correlation between percentage of red blush and total anthocyanin.


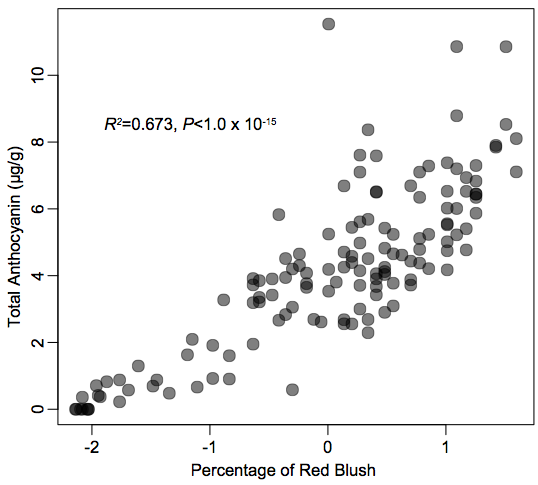


**Figure S6**: The correlation between browning and total phenolic content.


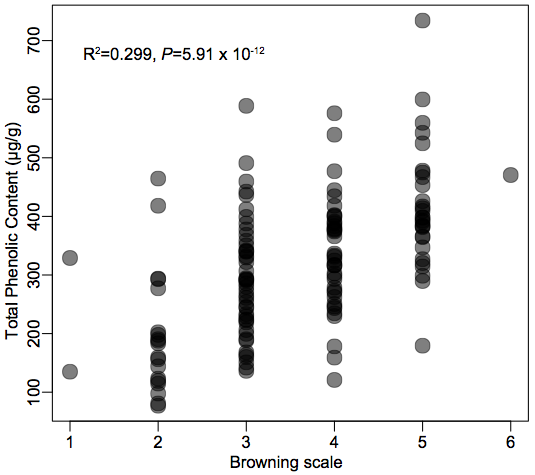


**Figure S7**: A boxplot showing the significant difference in quercitrin concentration between scab susceptible and scab resistant cultivars.


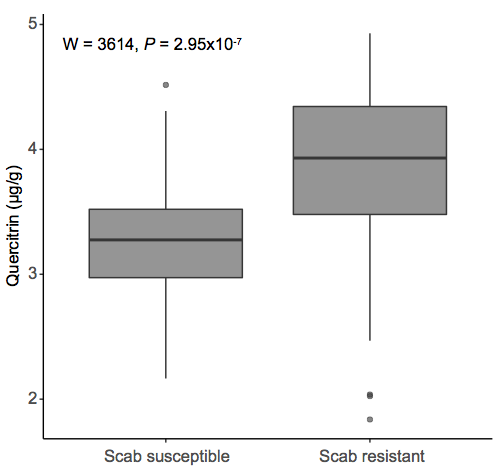


**Figure S8**: Manhattan plots for the 2014 phenotype data for all phenotypes not shown in the main manuscript.


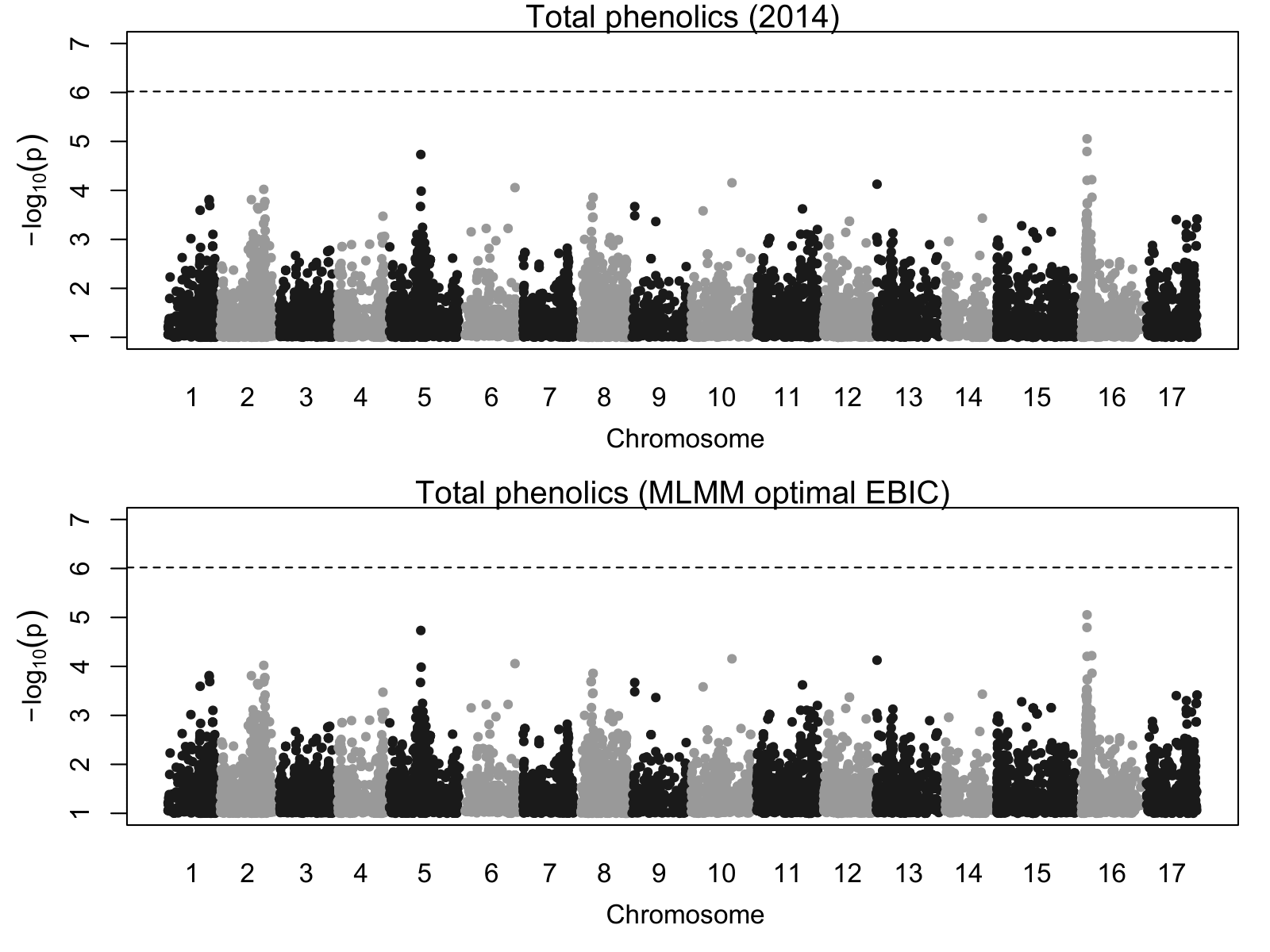

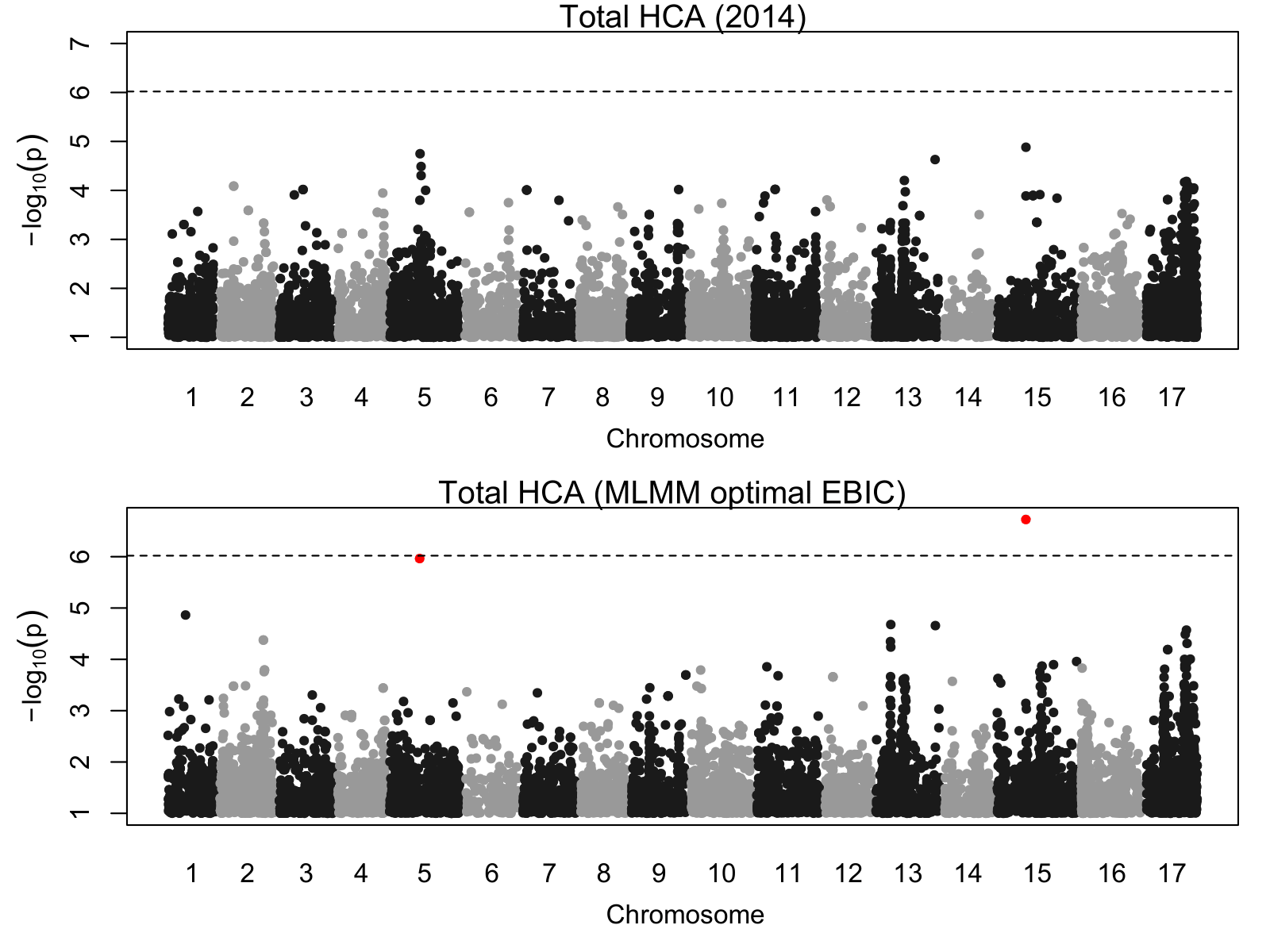

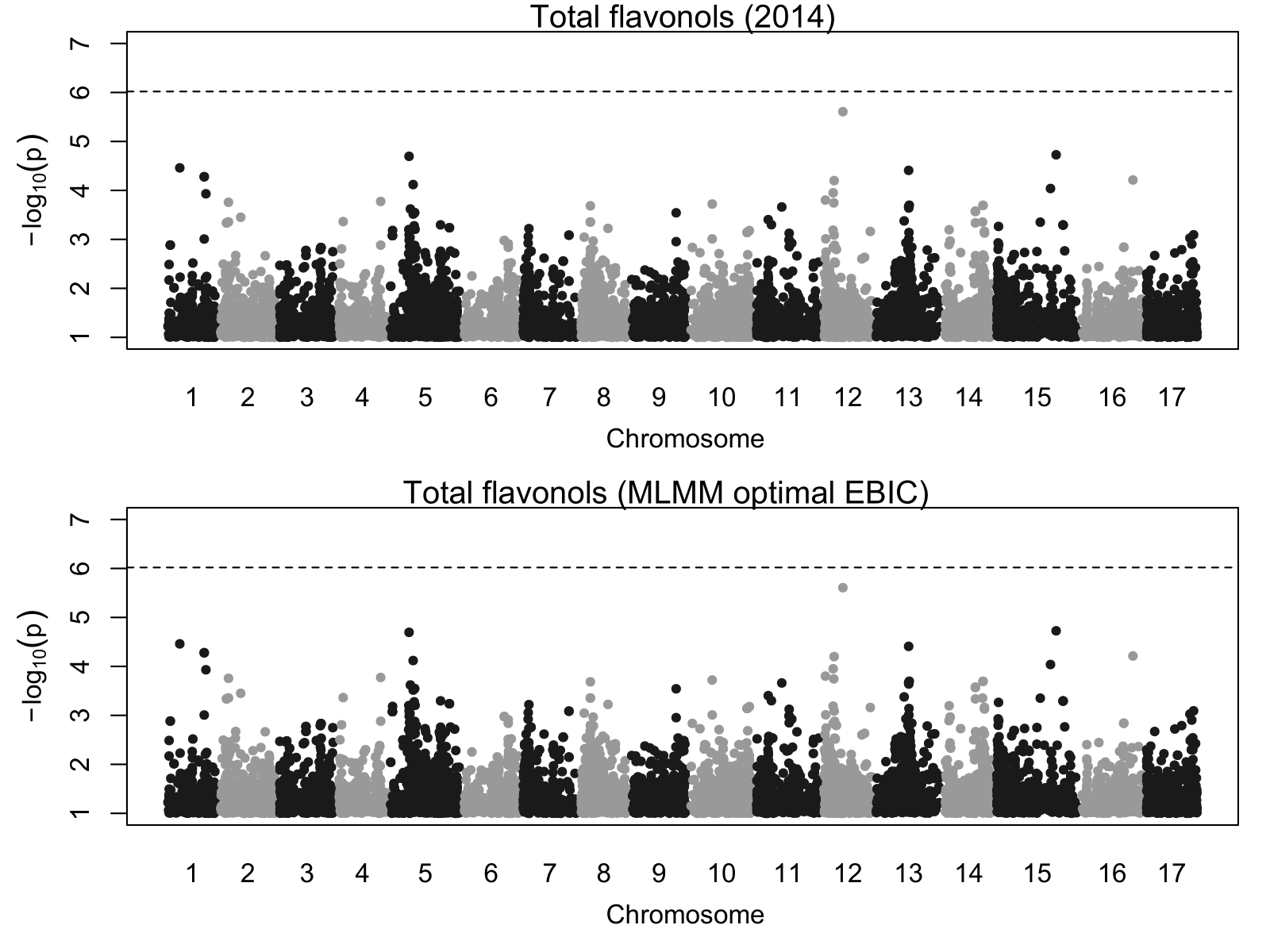

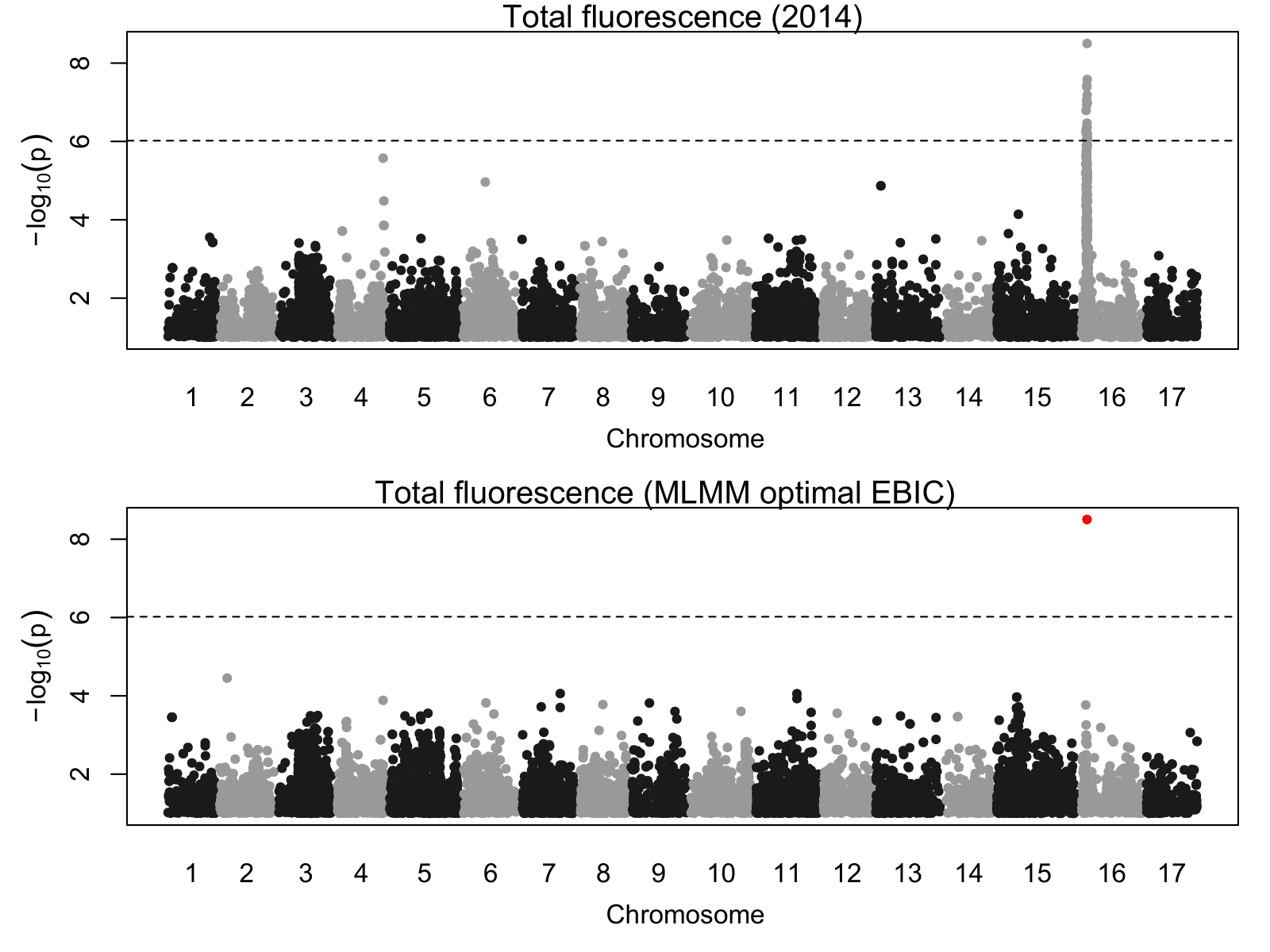

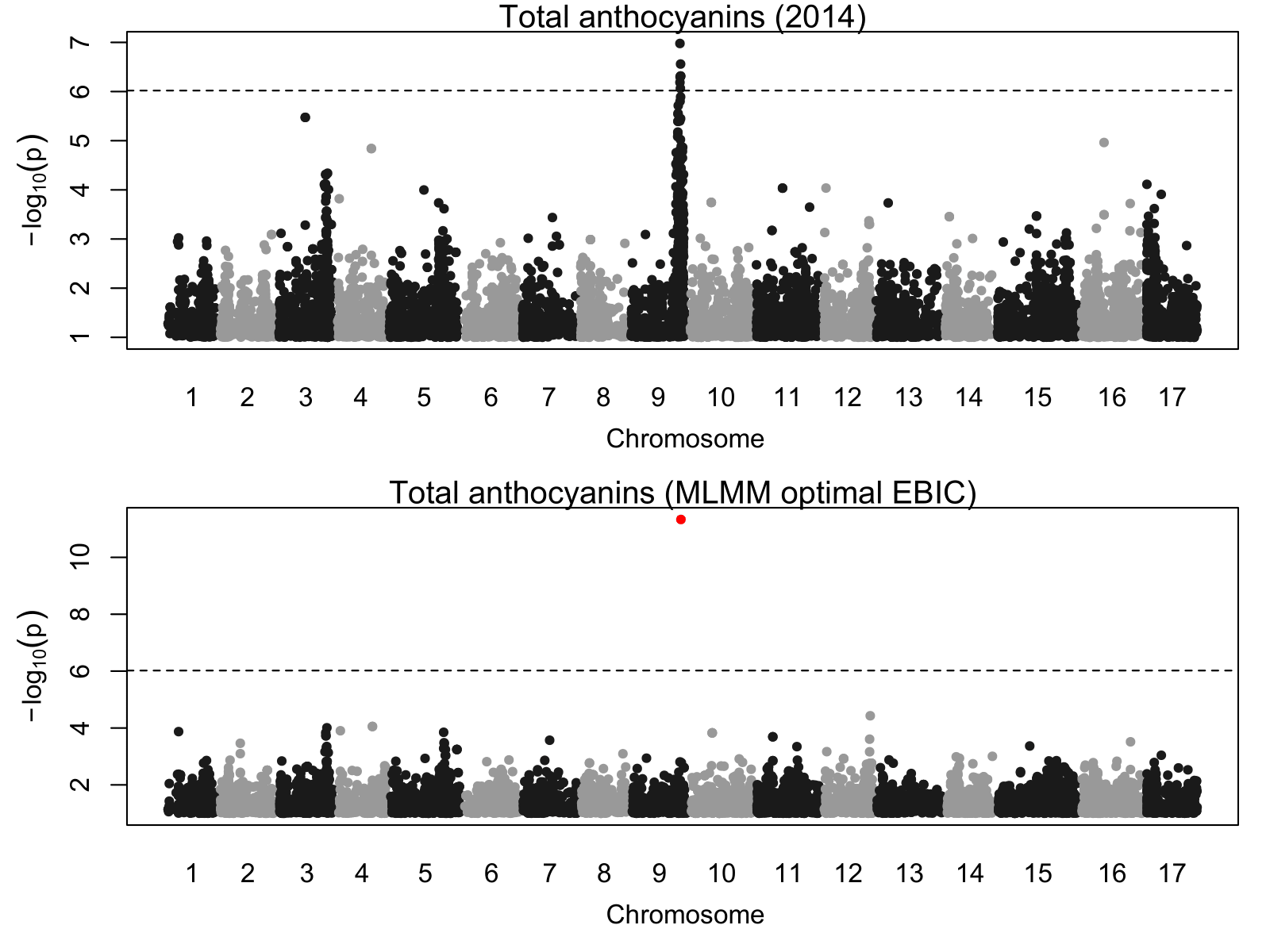

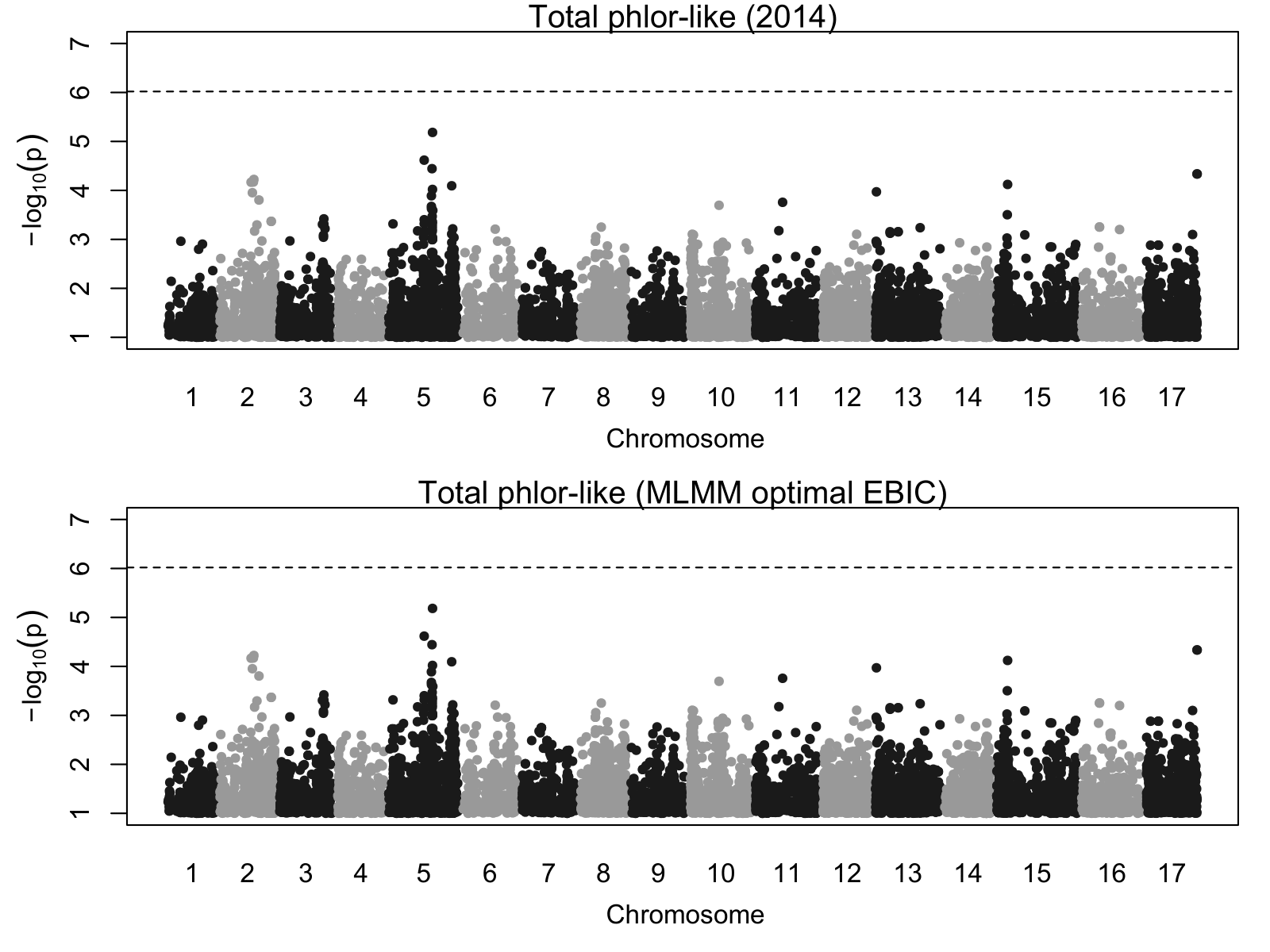

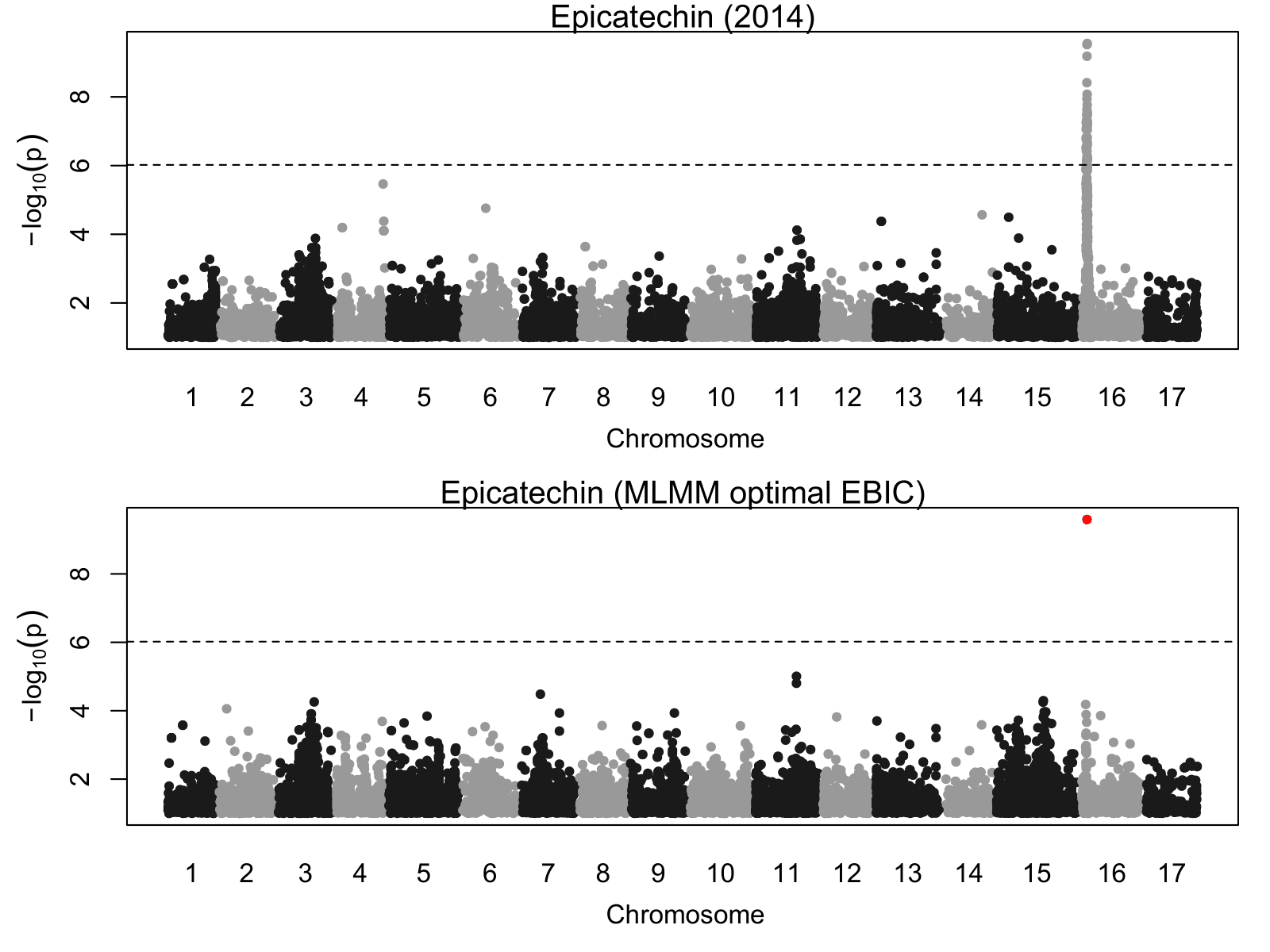

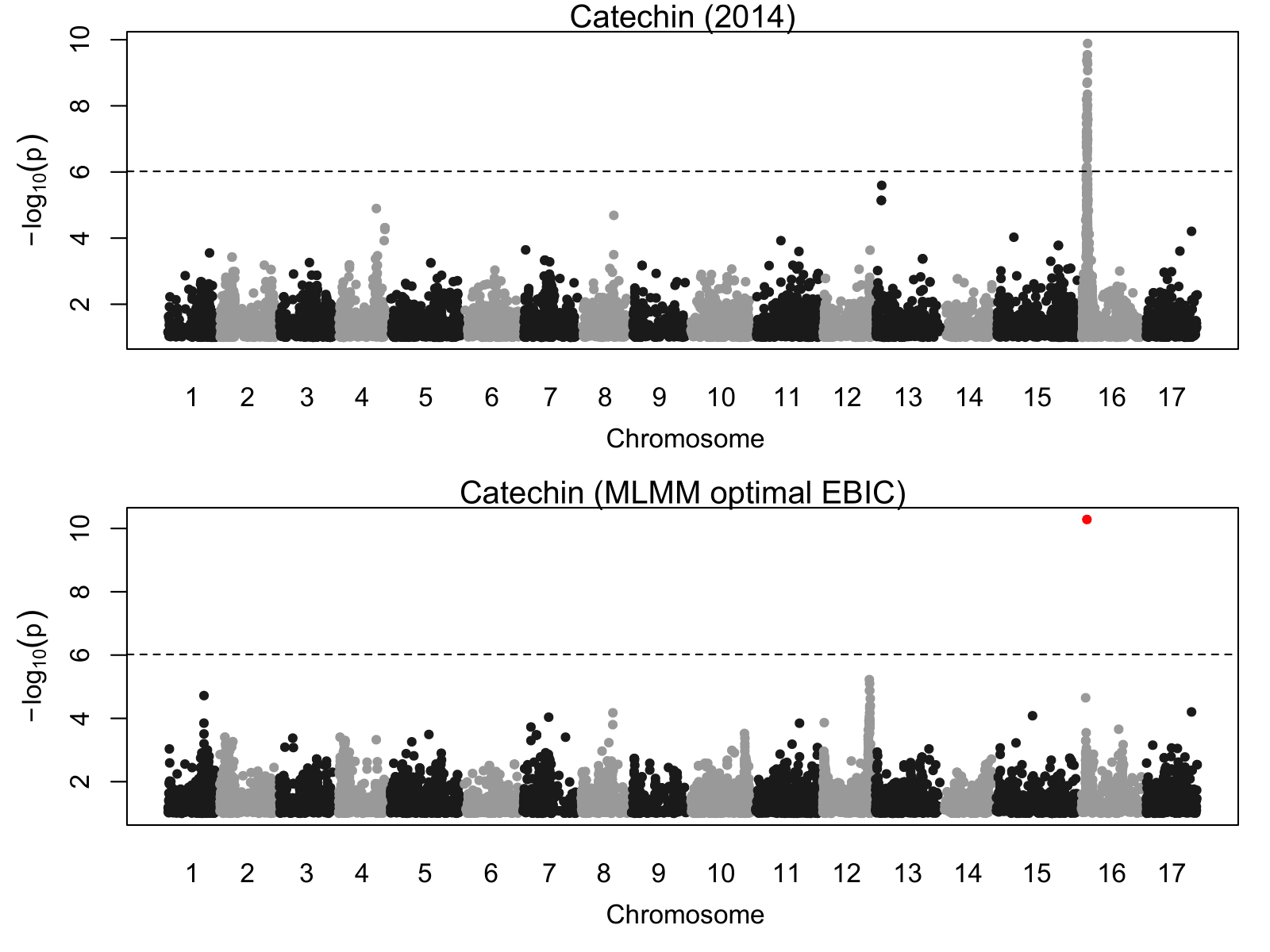

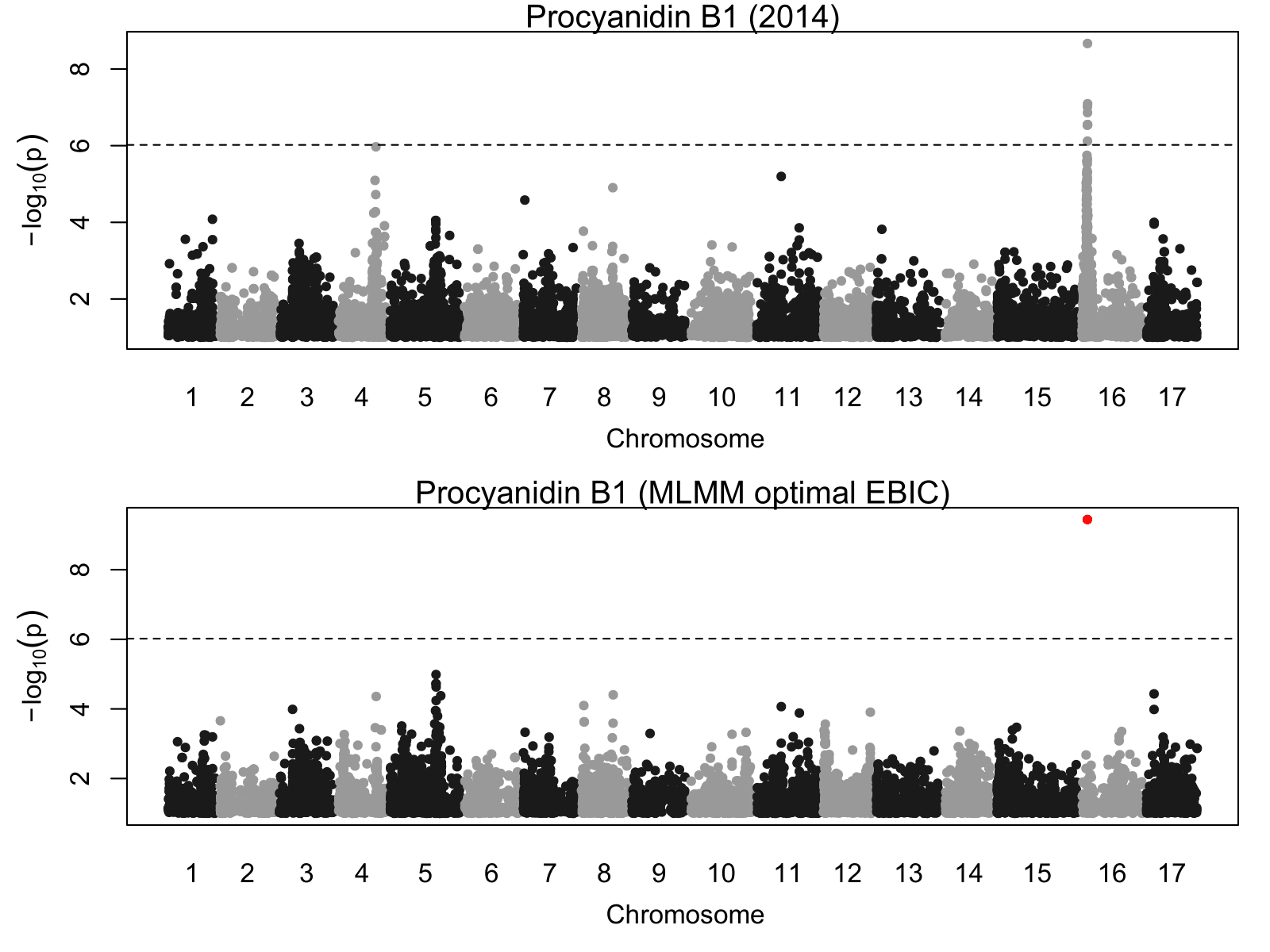

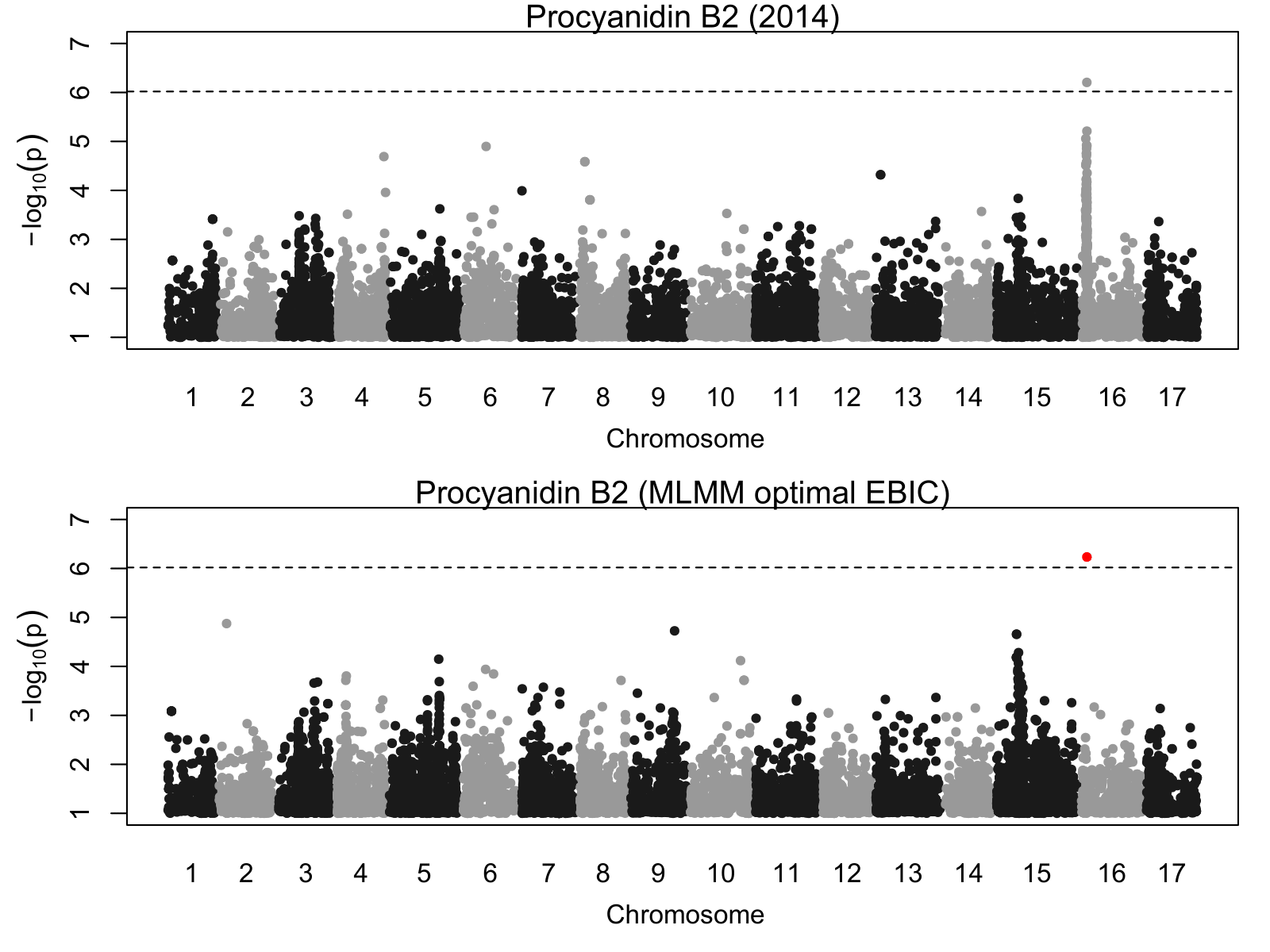

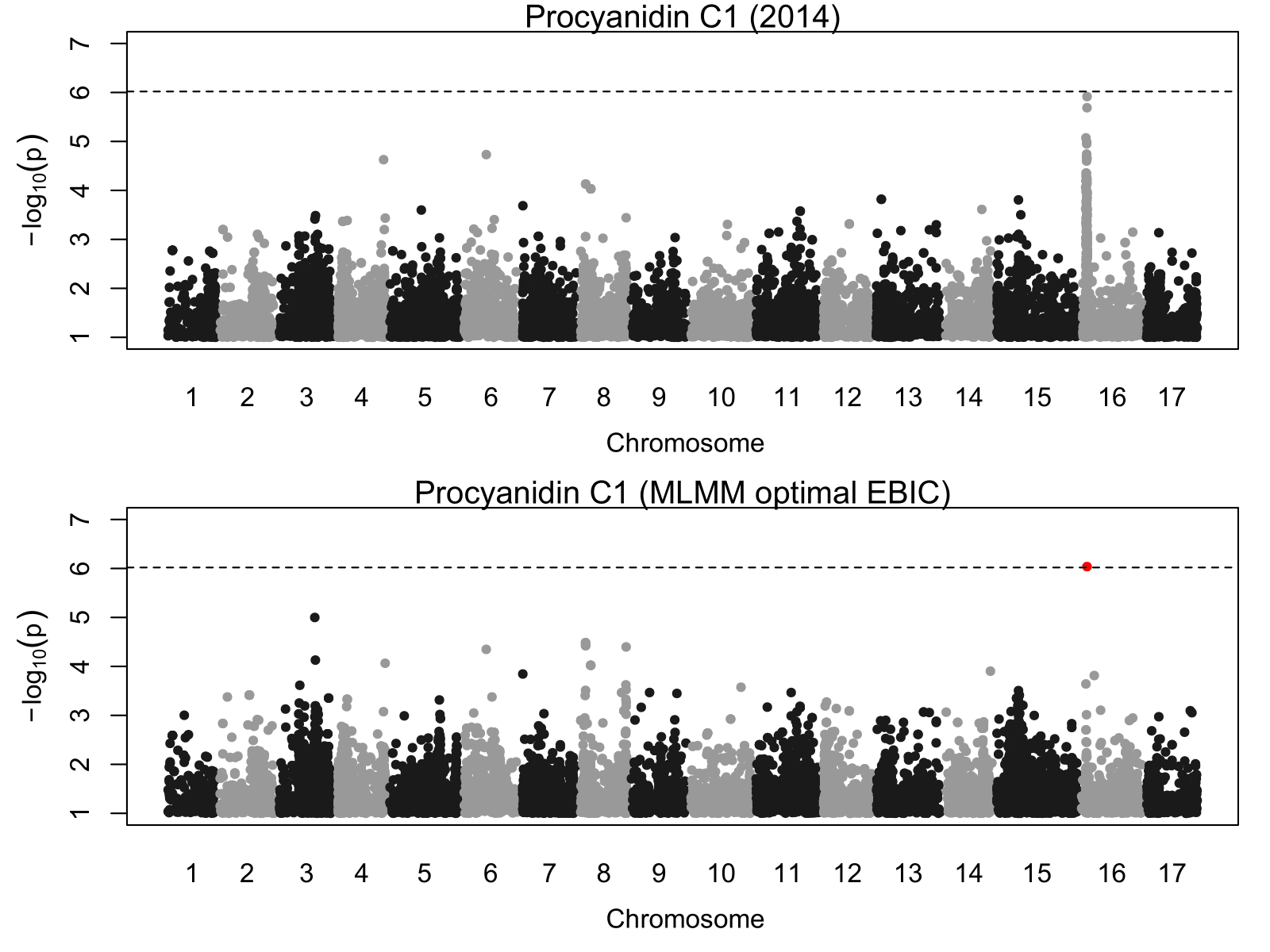

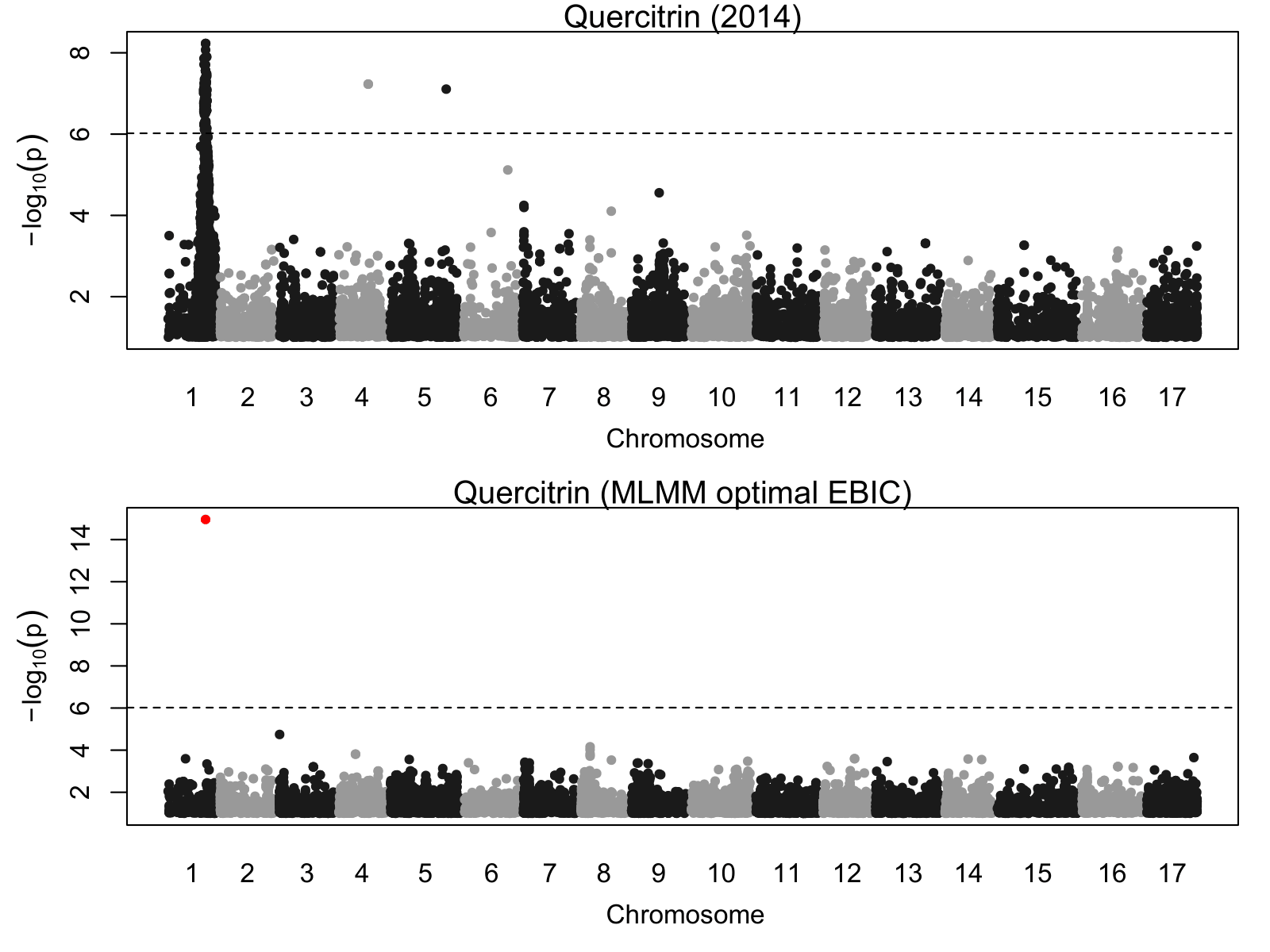

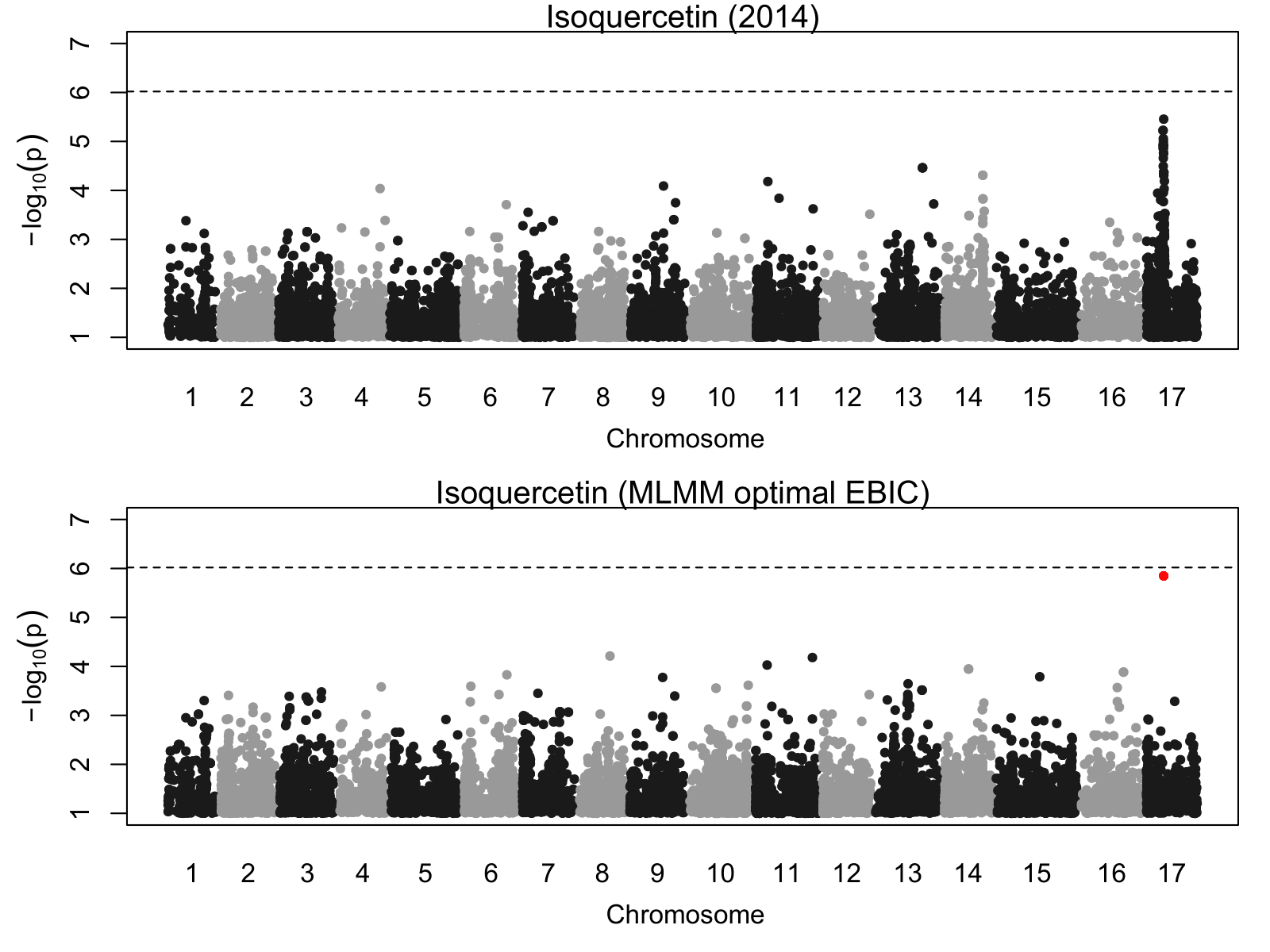

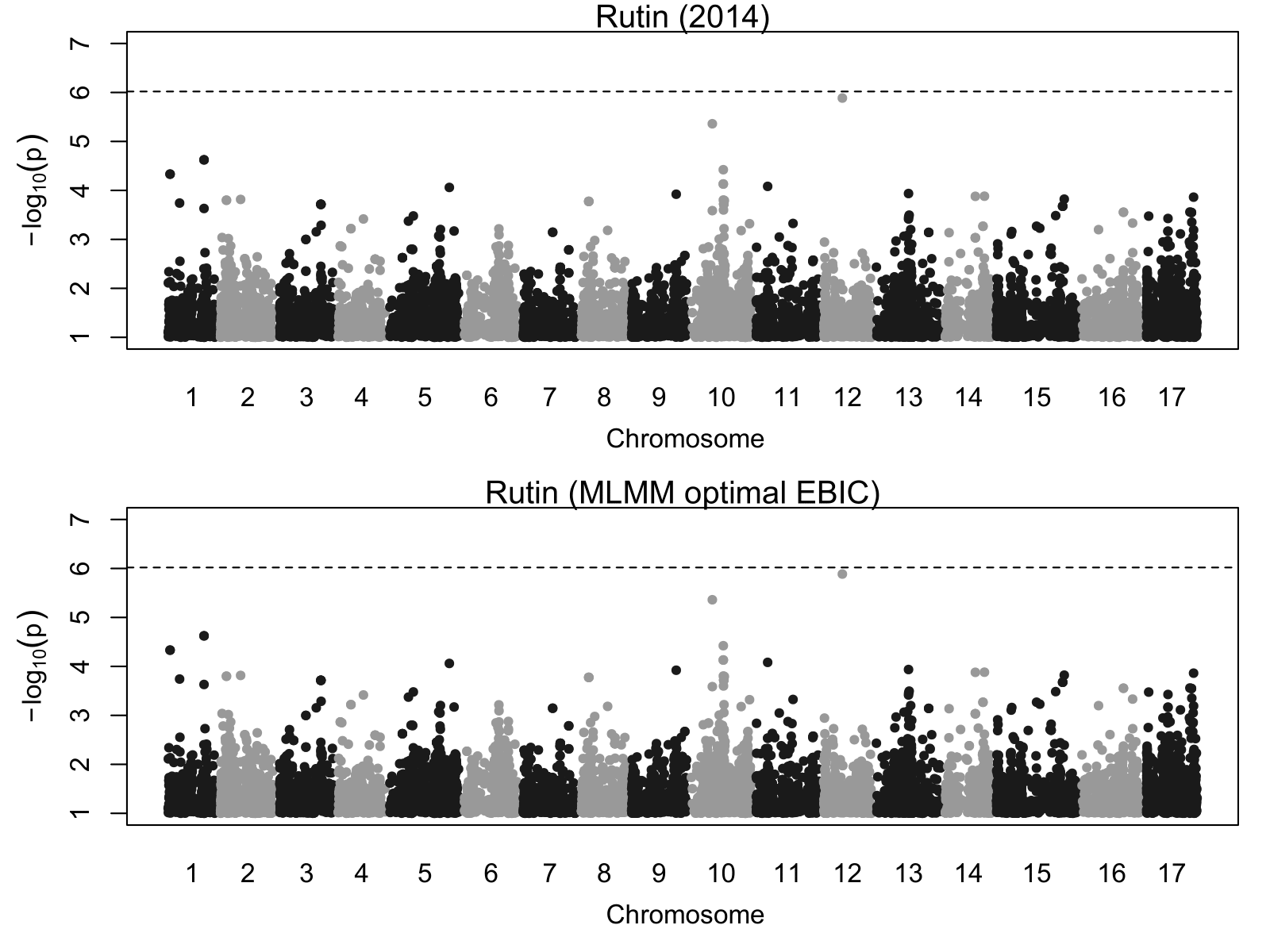

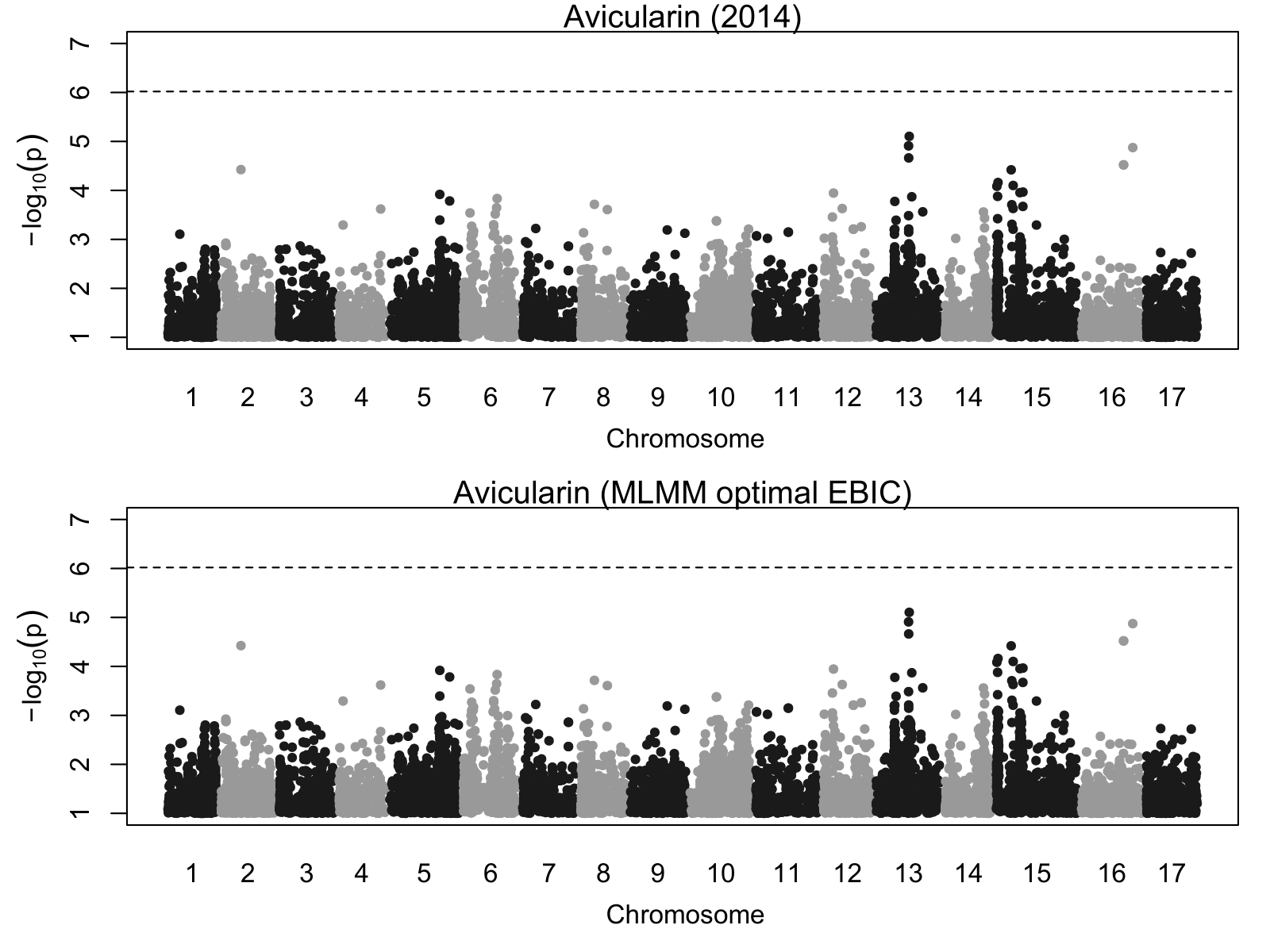

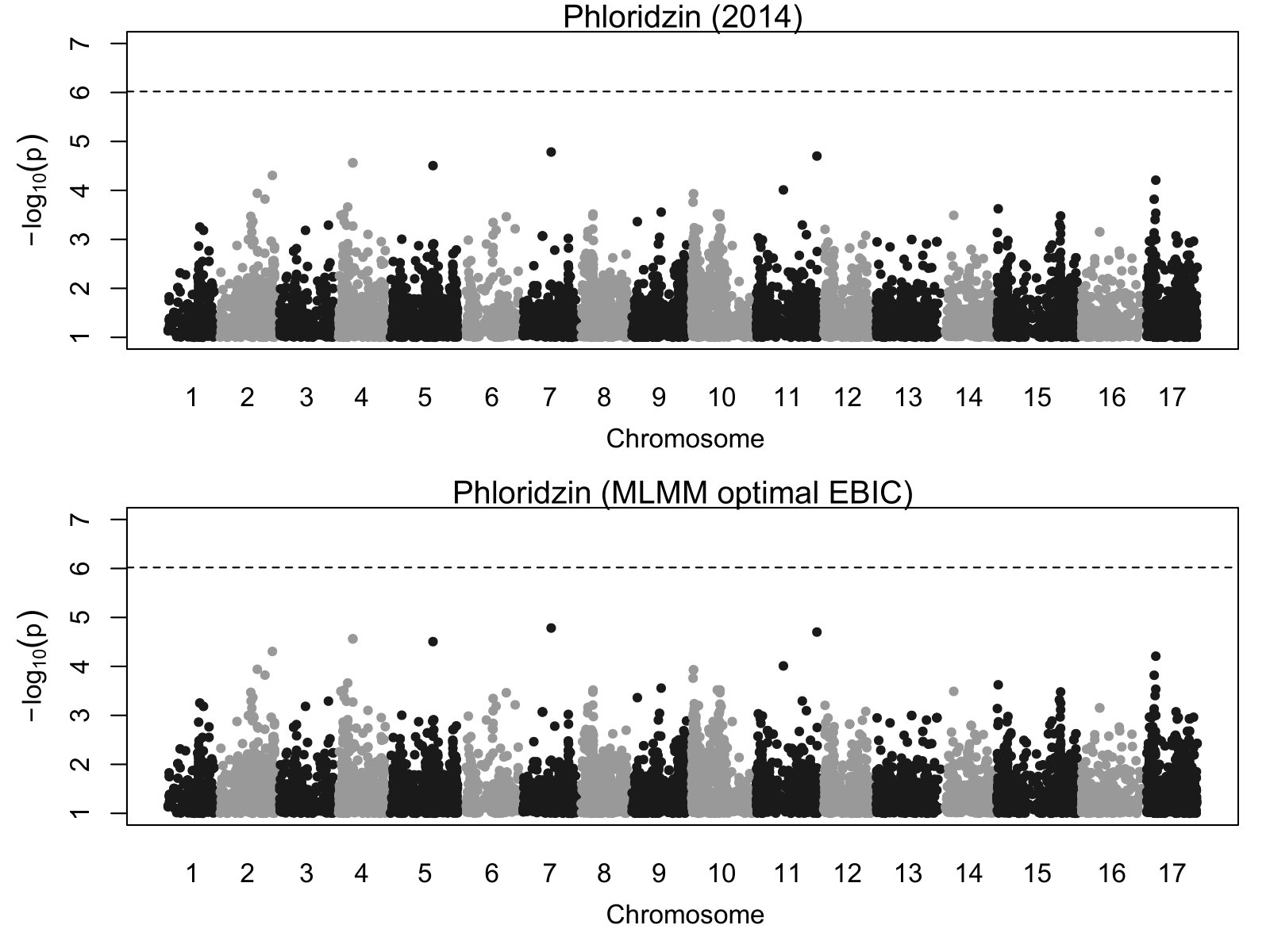

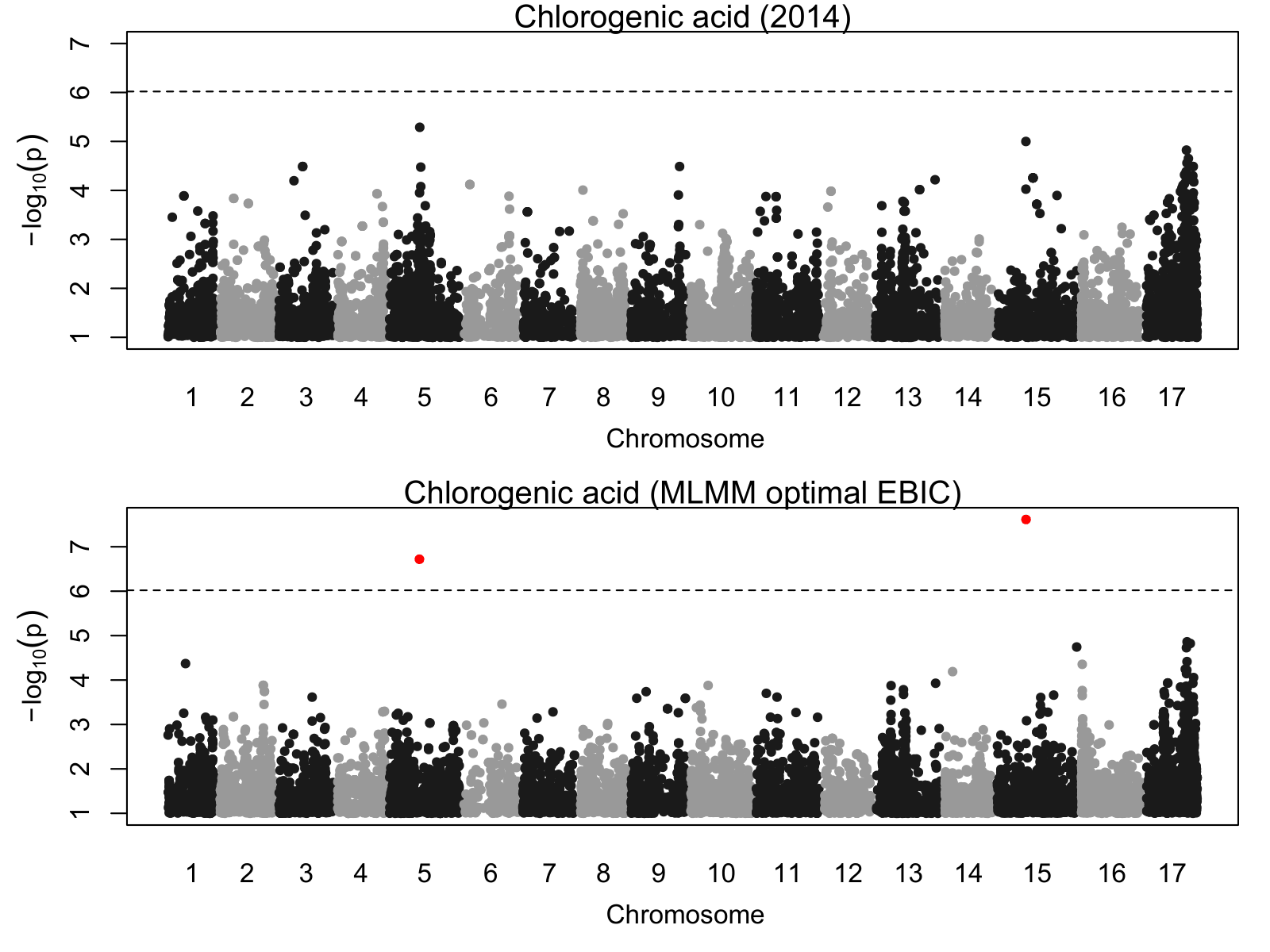

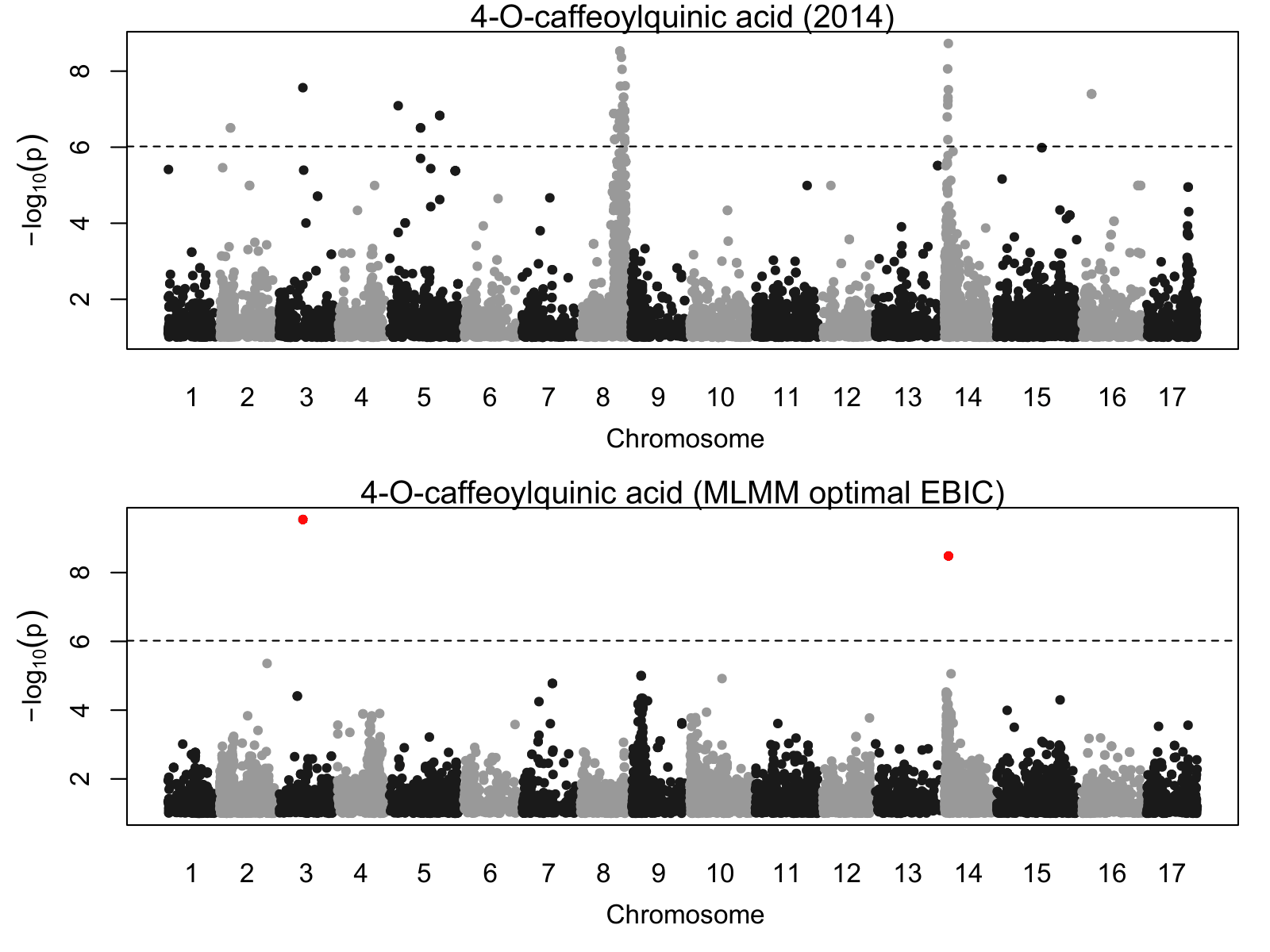

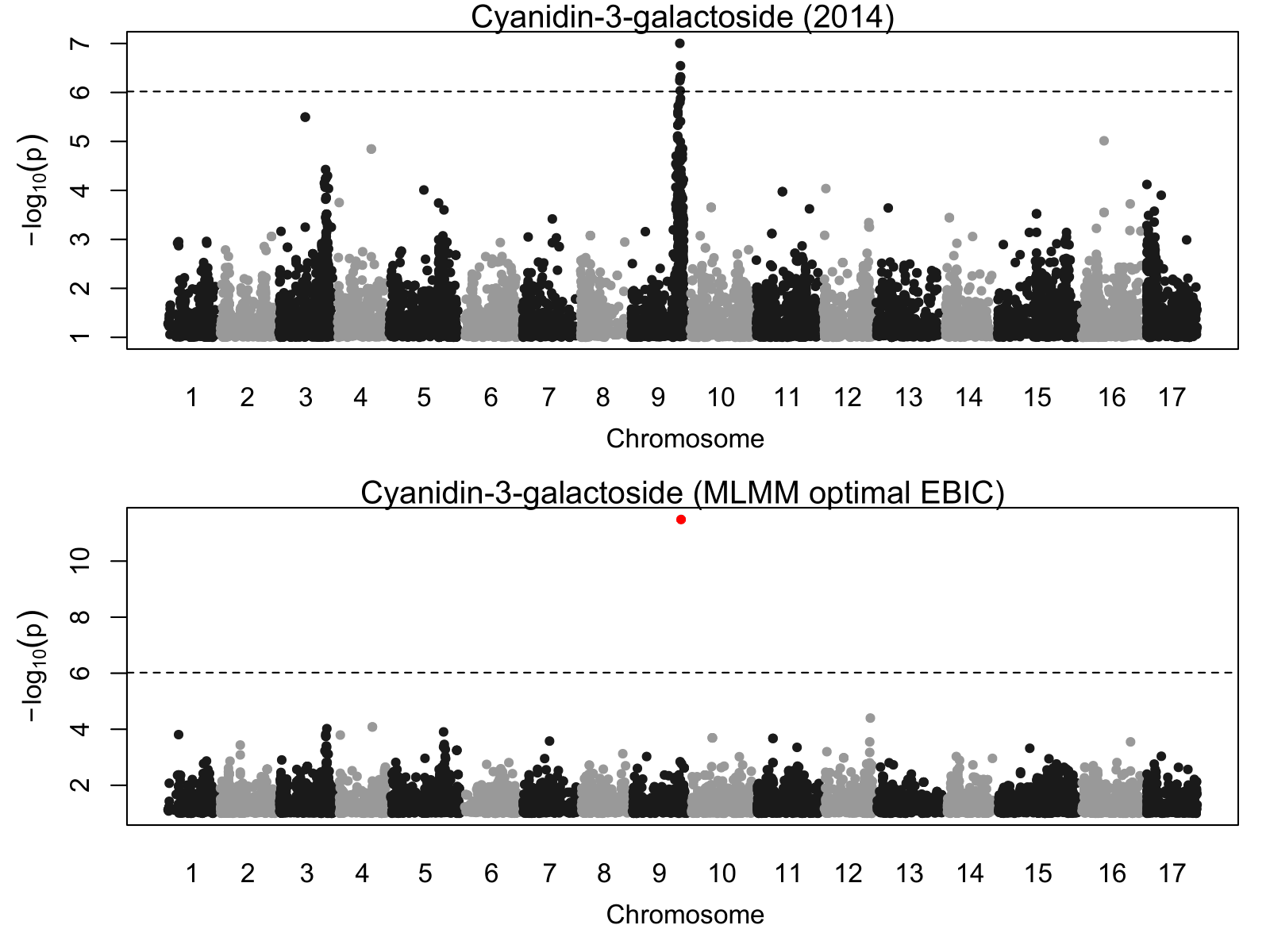


**Figure S9**: Manhattan plots of the GWAS results for the 2016 phenotype data.\


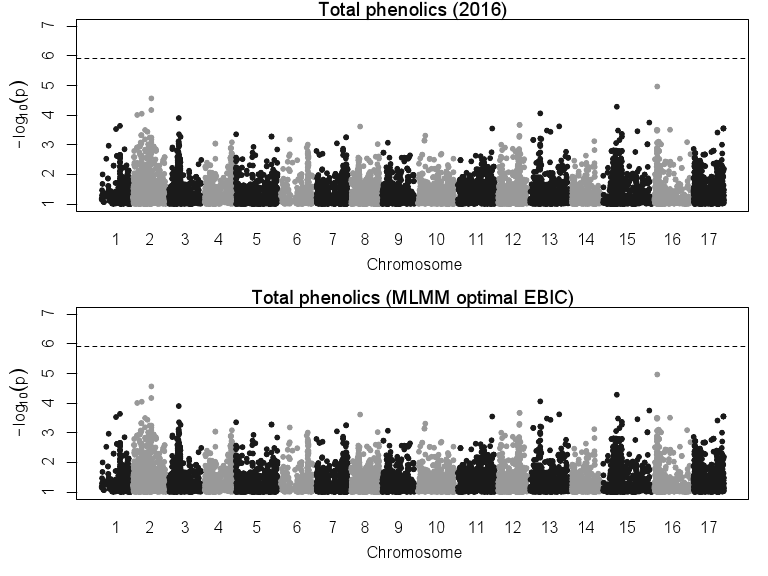


**
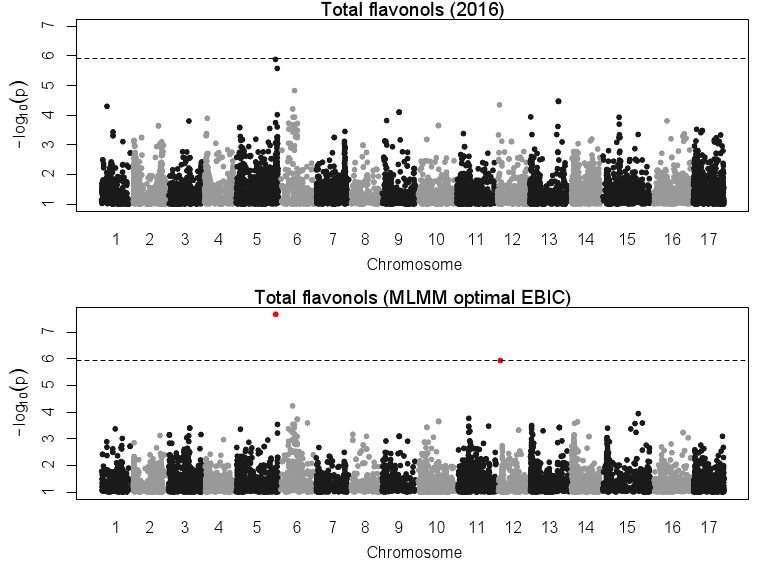

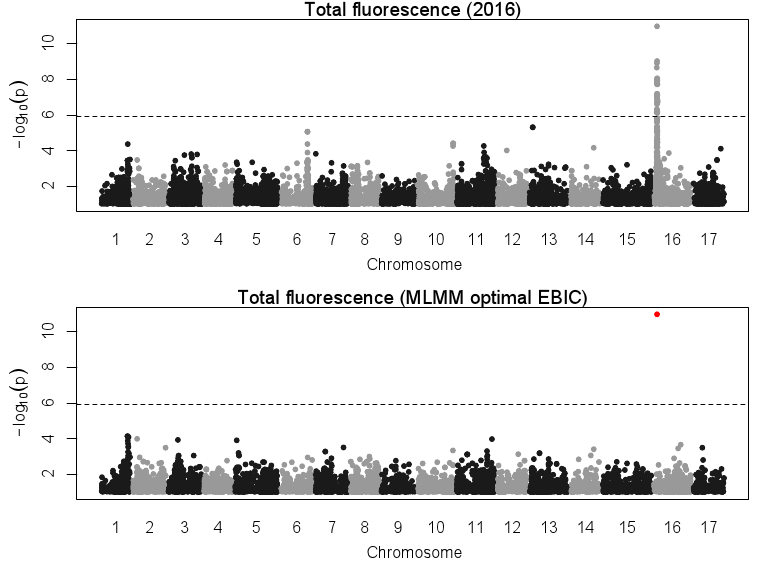

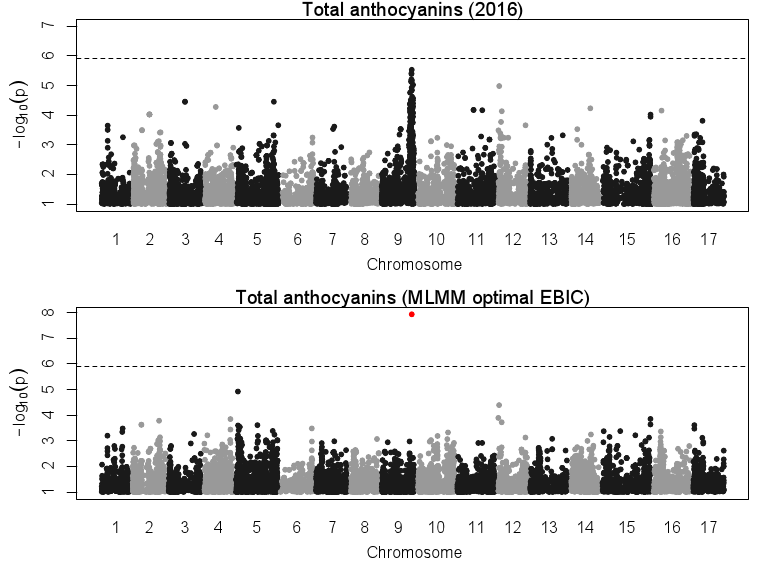

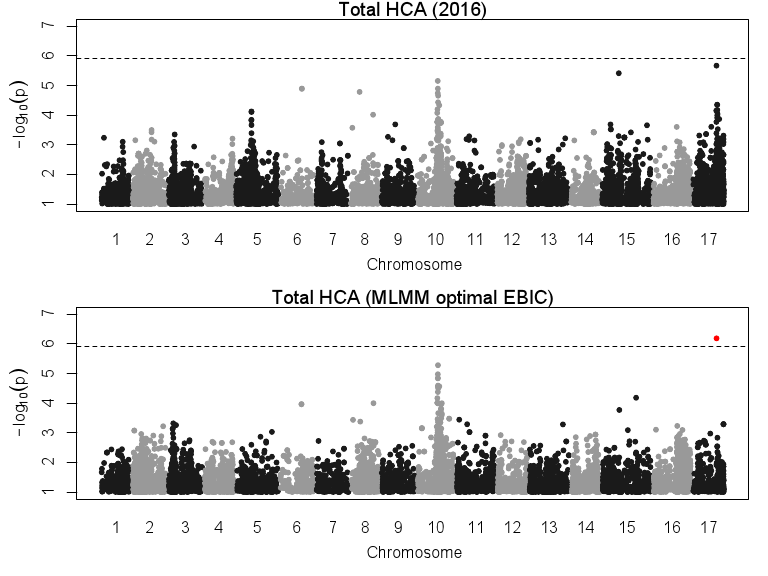
**

**
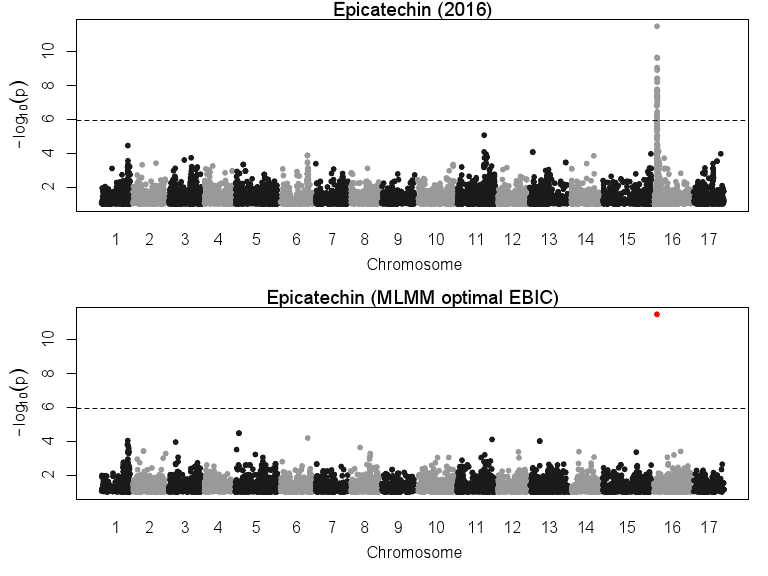

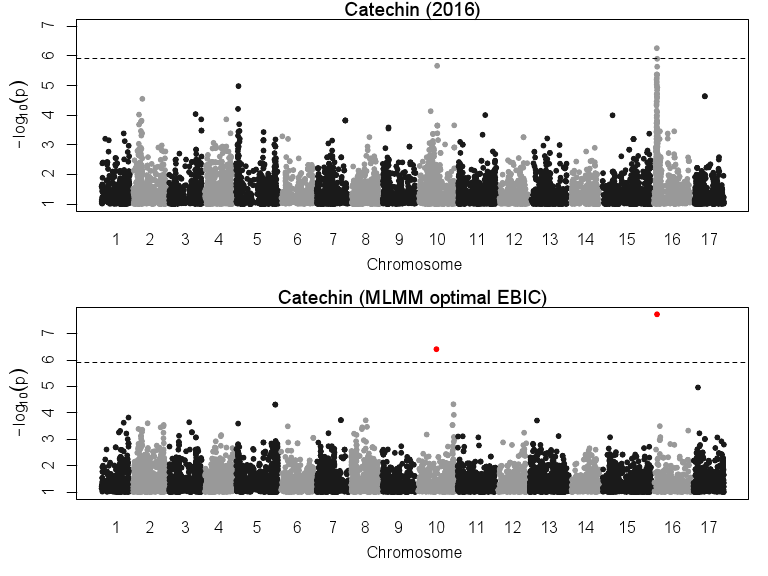

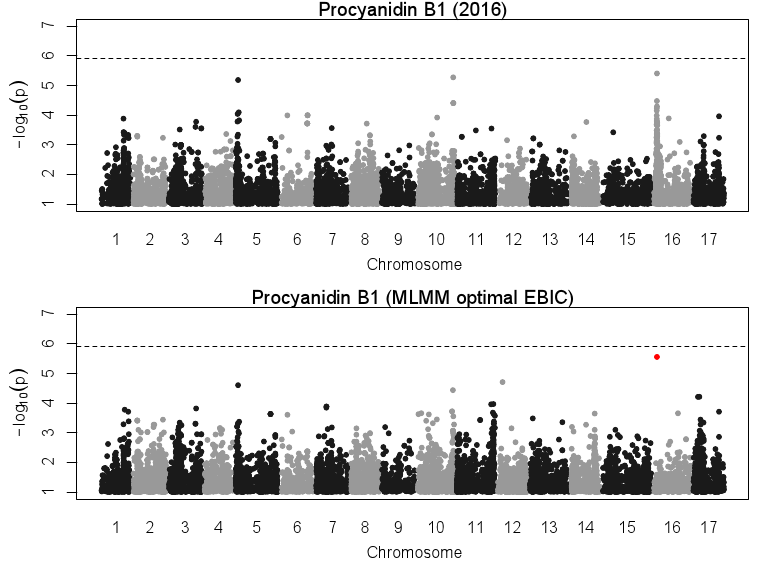

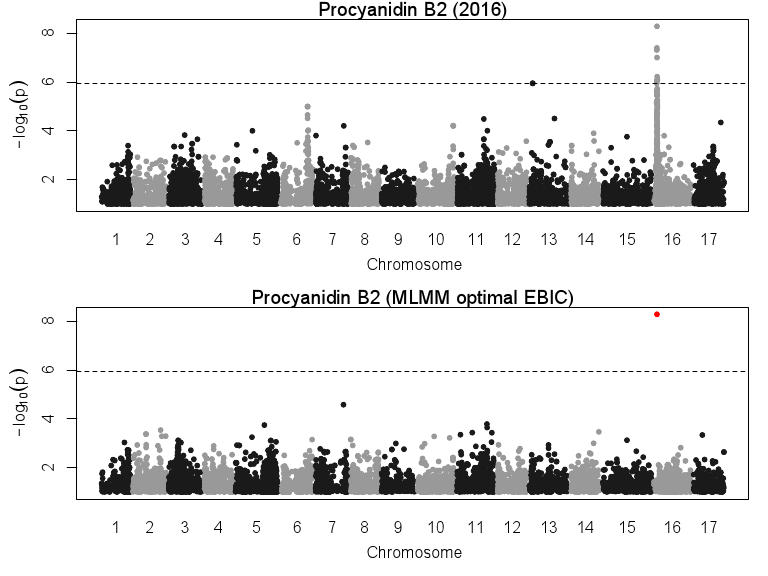

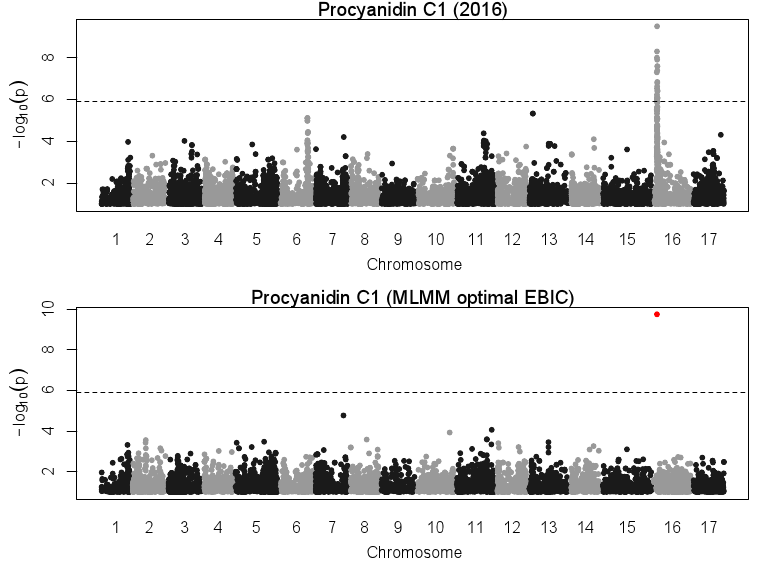

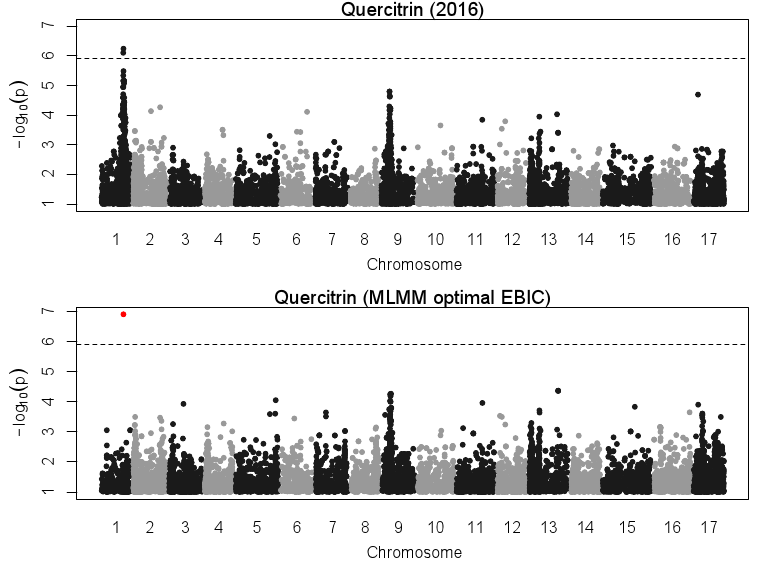

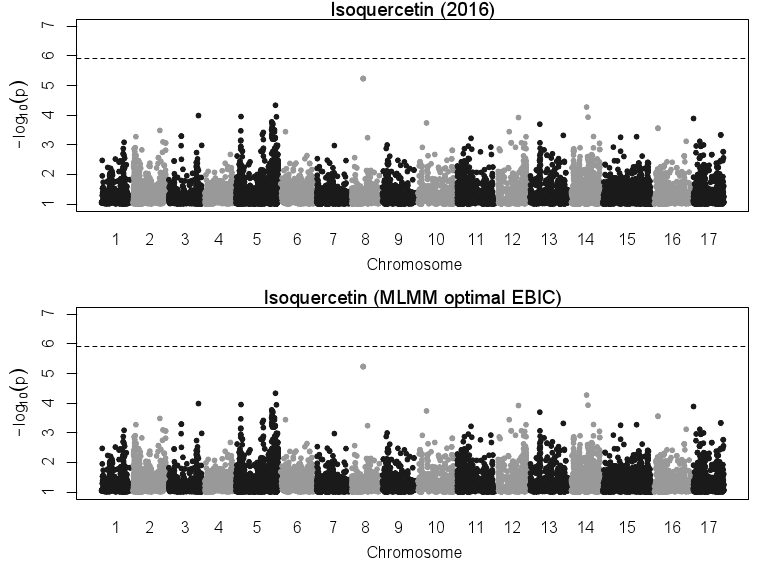

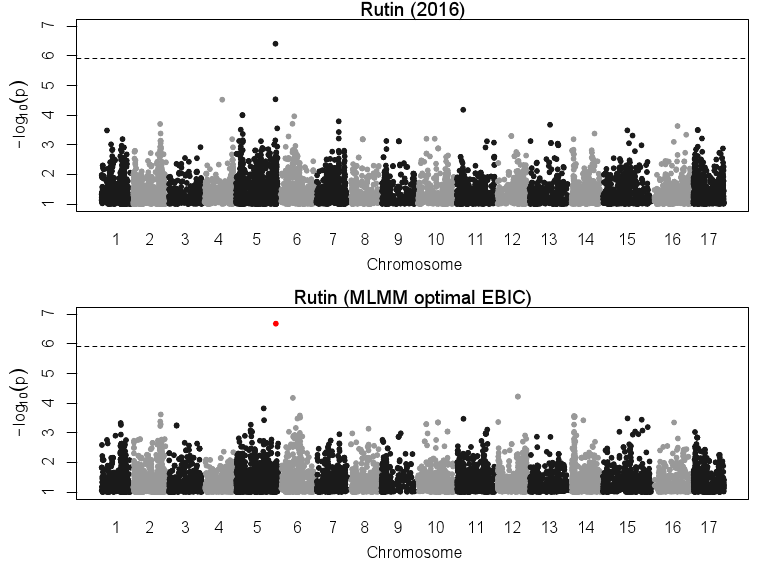

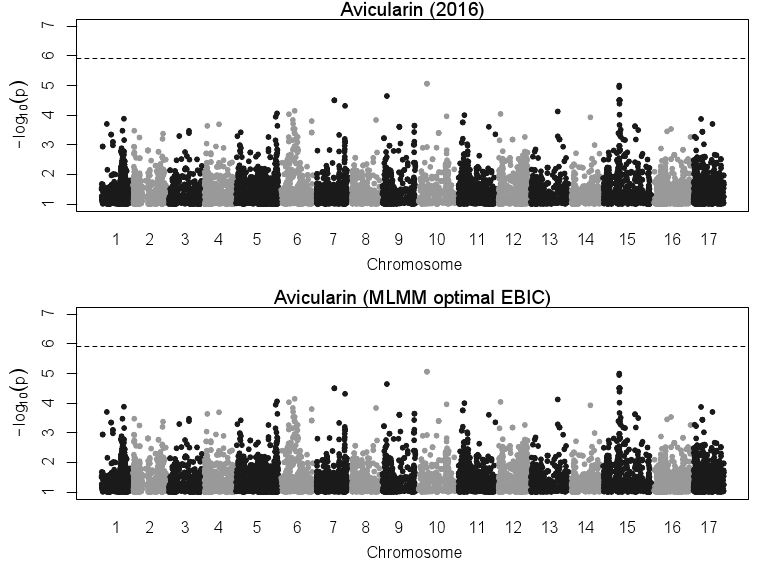

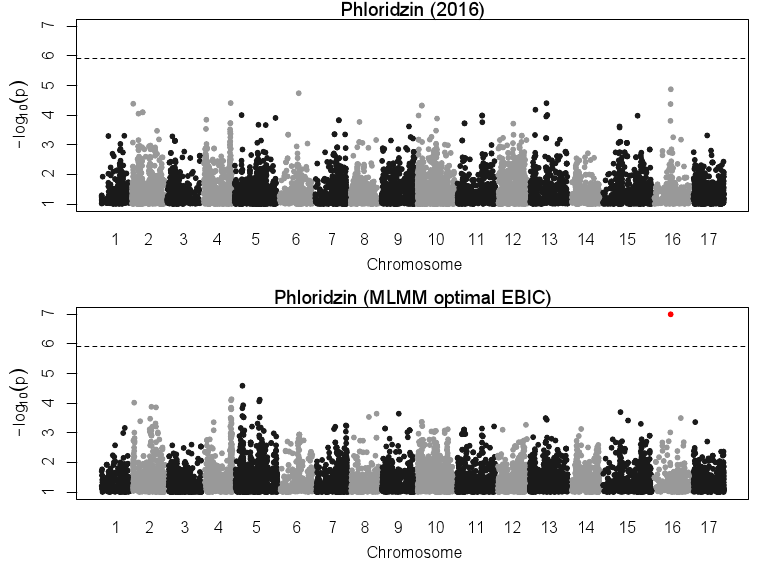

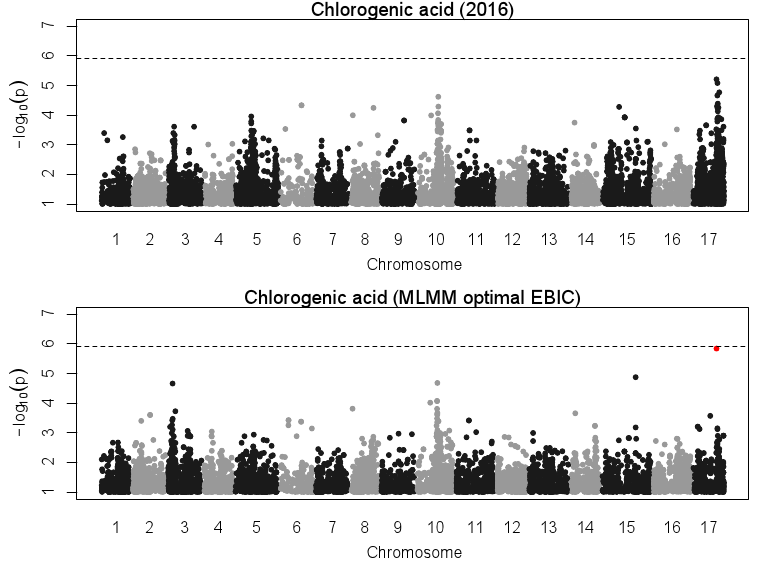

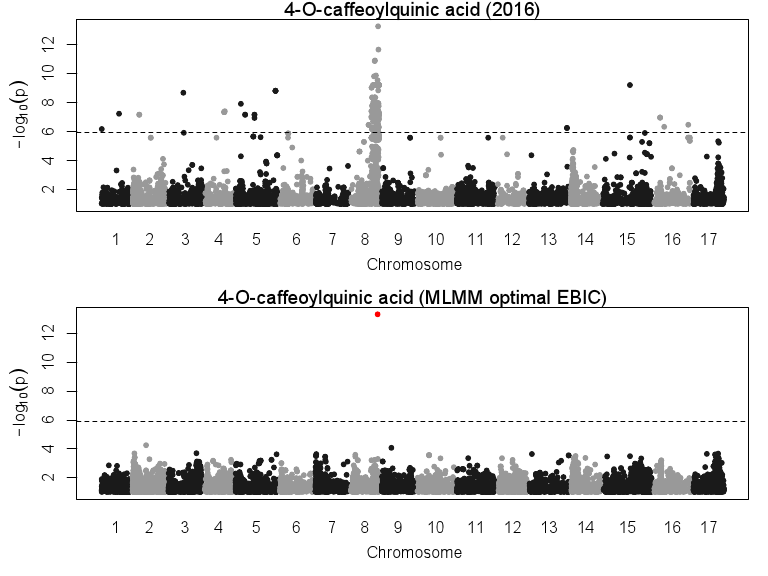

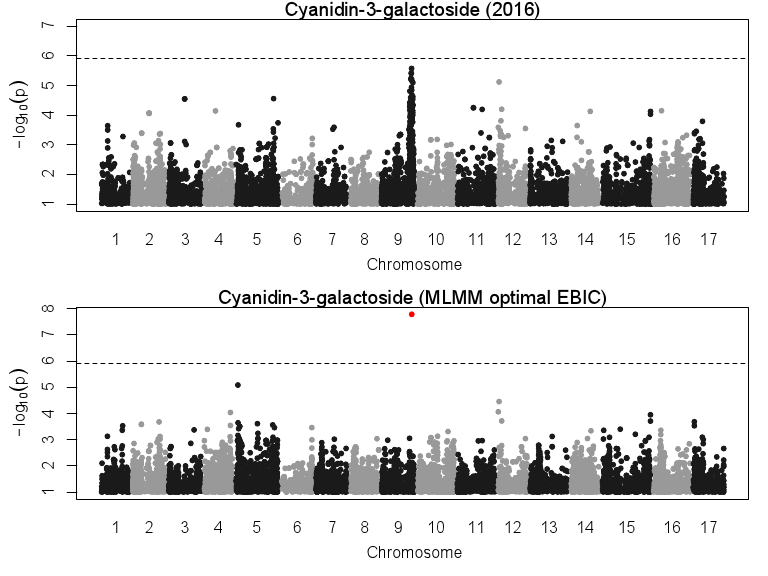
**

**Figure S10**: Estimates and standard deviations of genomic prediction (r) values for all polyphenols from the 2016 data.


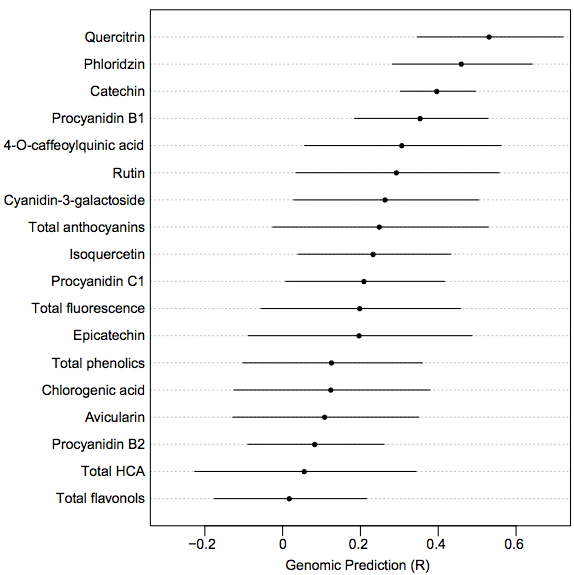


**Figure S11**: The correlation between years for genomic prediction accuracy.


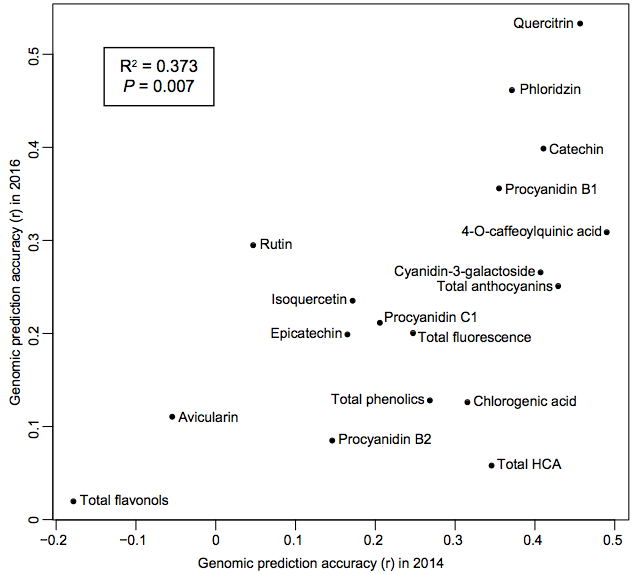


**Figure S12**: A Manhattan plot of chromosome 17 only for chlorogenic acid highlighting the position of the shikimate/quinate O-hydroxycinnamoyl transferase (HCT/HQT) genes.


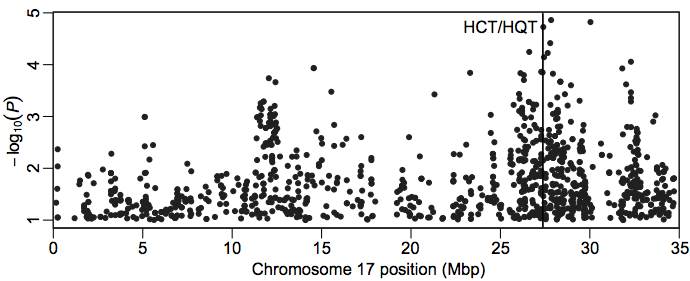


Figure S13: A Manhattan plot of chromosome 9 only for (A) cyanidin-3-galactoside and (B) total anthocyanins. The vertical line denotes the location of the *MYB10* gene.


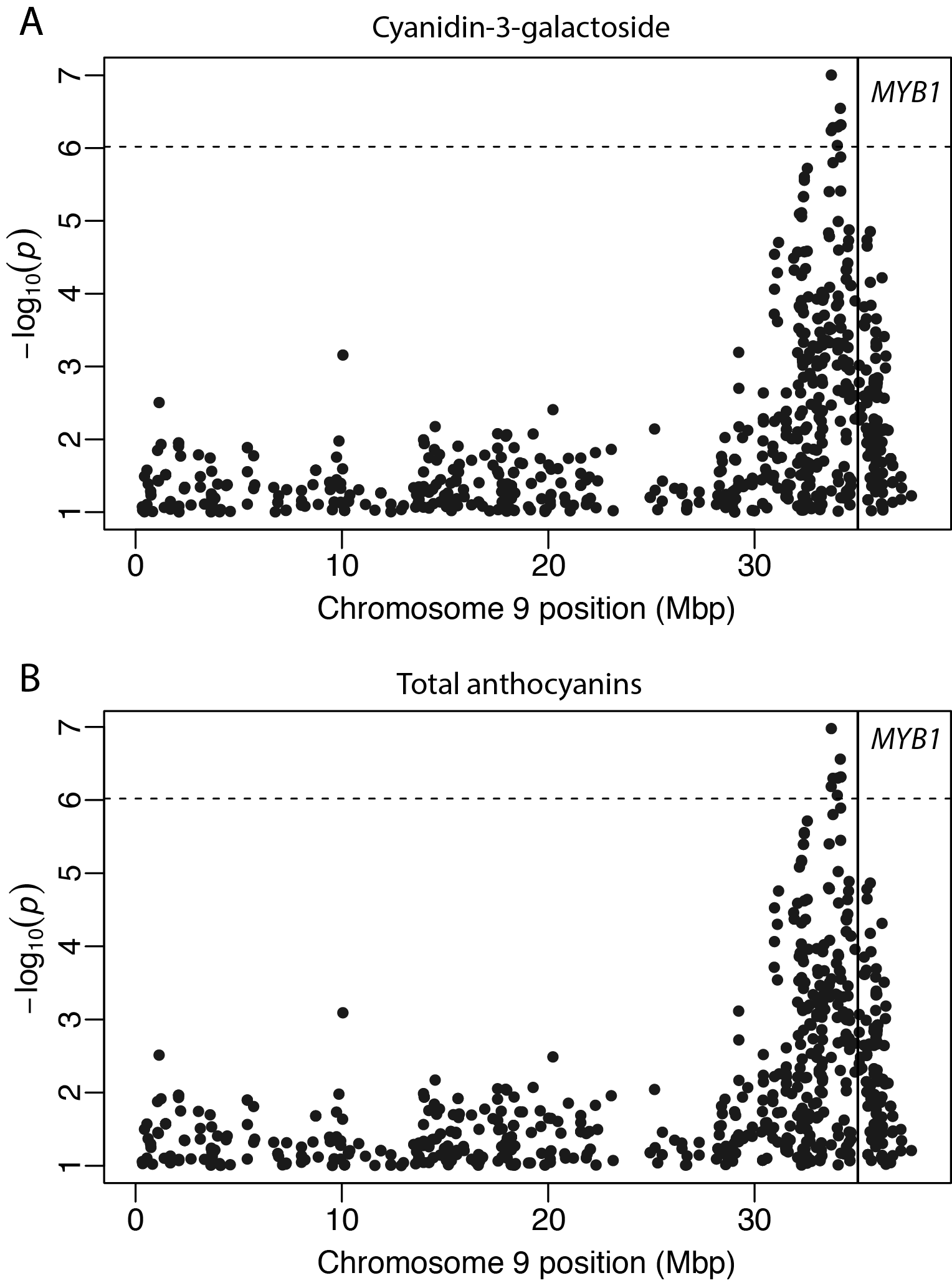

Supplement: Supplementary file 1 — Supplementary materials [file 41438_2019_190_MOESM1_ESM.docx]
